# Skeletal Muscle Transcriptomic Responses to Concurrent Training With Polyphenols in Ageing Adults: A Randomized Trial

**DOI:** 10.1002/jcsm.70391

**Published:** 2026-09-25

**Authors:** A. Guadalupe‐Grau, D. Gudra, D. Lismane, J. Raudeniece, G. Akuratere, K. Viksne, L. Gailite, B. Vilne, O. Pivovarova‐Ramich, M. Flensted‐Jensen, F. Dela, D. Reihmane

**Affiliations:** ^1^ GENUD Toledo Research Group, Faculty of Sport Sciences University of Castilla‐La Mancha Toledo Spain; ^2^ Centro de Investigación Biomédica en Red Fragilidad y Envejecimiento Saludable (CIBERFES) Instituto de Salud Carlos III Madrid Spain; ^3^ Grupo Mixto de Fragilidad y Envejecimiento Exitoso UCLM‐SESCAM (TEC2022‐007) Instituto de Investigación Sanitaria de Castilla‐La Mancha (IDISCAM), Junta de Comunidades de Castilla‐La Mancha (JCCM) Toledo Spain; ^4^ Scientific Laboratory of Molecular Genetics Riga Stradiņš University Riga Latvia; ^5^ Laboratory of Sports and Nutrition Research Riga Stradiņš University Riga Latvia; ^6^ Department of Human Physiology and Biochemistry Riga Stradiņš University Riga Latvia; ^7^ Bioinformatics Group Riga Stradiņš University Riga Latvia; ^8^ Department of Molecular Metabolism and Precision Nutrition German Institute of Human Nutrition Potsdam‐Rehbruecke Nuthetal Germany; ^9^ Department of Endocrinology and Metabolism Charité, Universitätsmedizin Berlin, Corporate Member of Freie Universität Berlin and Humboldt‐Universität zu Berlin Berlin Germany; ^10^ German Center for Diabetes Research (DZD) München‐Neuherberg Germany; ^11^ Xlab, Department of Biomedical Sciences, Faculty of Health Sciences University of Copenhagen Copenhagen Denmark

**Keywords:** ageing, polyphenols, skeletal muscle transcriptomics, training

## Abstract

**Background:**

Transcriptomic adaptations of ageing skeletal muscle to concurrent resistance training (RT) and high‐intensity interval training (HIIT) remain insufficiently characterized. We investigated the effects of a 12‐week concurrent RT + HIIT intervention on the vastus lateralis transcriptome in middle‐aged and older adults and whether polyphenol supplementation modulated these responses.

**Methods:**

Thirty‐eight adults (55–70 years; 17 polyphenol [PP], 21 placebo [PLA]) with paired skeletal muscle RNA‐sequencing data participated in a randomized, double‐blind trial. Participants completed a 30‐day supplementation phase followed by a 12‐week supervised RT + HIIT programme with continued supplementation. Muscle biopsies were obtained before (PRE) and after (POST) training. Differential expression analyses were adjusted for age, sex and muscle fibre type composition. Differential expression was assessed using FDR ≤ 0.05, with |log_2_FC| > 1 applied as an additional effect‐size criterion.

**Results:**

Baseline transcriptomic differences between PP and PLA groups were minimal, with three FDR‐significant transcripts, of which only *HMOX1* additionally exceeded |log_2_FC| > 1 (FDR = 0.011; log_2_FC = 1.16). The group‐by‐time interaction analysis identified 14 FDR‐significant transcripts, of which eight additionally exceeded |log_2_FC| > 1; these signals were characterized by low or sparse expression and high inter‐individual variability, without a coherent supplementation‐related transcriptional response. In the pooled paired PRE–POST analysis, 60 transcripts reached FDR significance, of which seven additionally exceeded |log_2_FC| > 1, including upregulation of *CLLU1* (log_2_FC = 3.10; FDR = 0.0155), *PRND* (log_2_ log_2_FC = 2.60; FDR = 0.02) and *LOX*, and downregulation of *MYH1* (log_2_FC = −1.3; FDR = 0.0001). Most FDR‐significant transcripts showed relatively small estimated effect sizes, and functional enrichment analyses did not identify robust pathway‐level adaptations.

**Conclusions:**

In middle‐aged and older adults, 12 weeks of concurrent RT + HIIT induced a predominantly low‐magnitude skeletal muscle transcriptional response. Selected transcript‐level changes were consistent with processes related to extracellular matrix remodelling and contractile regulation; however, the absence of robust pathway‐level enrichment limits conclusions regarding coordinated biological pathway adaptations. Polyphenol supplementation did not meaningfully influence the transcriptional response to exercise training.

## 1 Introduction

Ageing is accompanied by progressive declines in skeletal muscle mass, strength and functional capacity, which contribute to frailty, loss of independence and increased risk of disability [1]. At the cellular level, these alterations arise from the convergence of multiple biological processes that align with key hallmarks of ageing, including deductions in mitochondrial content and oxidative capacity, dysregulated nutrient sensing and anabolic signalling, increased oxidative stress, chronic low‐grade inflammation and impaired neuromuscular integrity [2]. The integration of these hallmarks is reflected in coordinated alterations in gene expression, indicative of progressive transcriptional dysregulation involving metabolic, inflammatory and extracellular matrix–related pathways that collectively underlie age‐related deterioration in skeletal muscle structure and function [3].

Exercise training is one of the most effective strategies to counteract this age‐related deterioration in skeletal muscle. In particular, combined resistance training (RT) and high‐intensity interval training (HIIT) improve muscle strength, body composition, cardiorespiratory fitness and metabolic health in older adults [4, 5, 6]. In a previous analysis from the same randomized trial, concurrent RT–HIIT enhanced skeletal muscle mitochondrial respiratory capacity and reduced H_2_O_2_ emission, indicating favourable adaptations in mitochondrial bioenergetics and redox homeostasis [6]. Emerging evidence from multi‐omics approaches indicates that distinct exercise modalities, including RT and HIIT, induce coordinated yet modality‐specific molecular adaptations in skeletal muscle, involving pathways related to extracellular matrix remodelling, metabolism and neuromuscular function [7]. However, it remains unclear whether concurrent exercise paradigms combining RT and HIIT elicit consistent transcriptome‐wide remodelling in ageing human skeletal muscle.

Nutritional factors may further modulate molecular responses to exercise by influencing signalling pathways that regulate gene expression. Polyphenol‐rich supplements have been proposed to interact with redox‐sensitive and inflammatory pathways, including mechanisms linked to mitochondrial function and cellular stress responses [8]. However, evidence supporting their capacity to enhance exercise‐induced skeletal muscle adaptations in older adults remains inconsistent. In the same cohort, polyphenol supplementation did not augment the improvements in muscle mass, strength and aerobic capacity induced by RT–HIIT, despite modest effects on circulating metabolic and antioxidant‐related markers [5]. These findings raise the possibility that any modulatory effects of polyphenols may occur at the molecular level, potentially influencing transcriptional responses without translating into measurable physiological benefits. Accordingly, the aim of the present study was to characterize the skeletal muscle transcriptomic response to a 12‐week concurrent RT–HIIT intervention in middle‐aged and older adults and to determine whether polyphenol supplementation modulates these molecular adaptations. We hypothesized that concurrent RT–HIIT would induce transcriptomic remodelling across pathways involved in skeletal muscle adaptation, including processes related to extracellular matrix organization, metabolism and inflammation. We further hypothesized that any modulatory effect of polyphenol supplementation would be limited.

## 2 Material and Methods

### 2.1 Study Design

**FIGURE 1:**
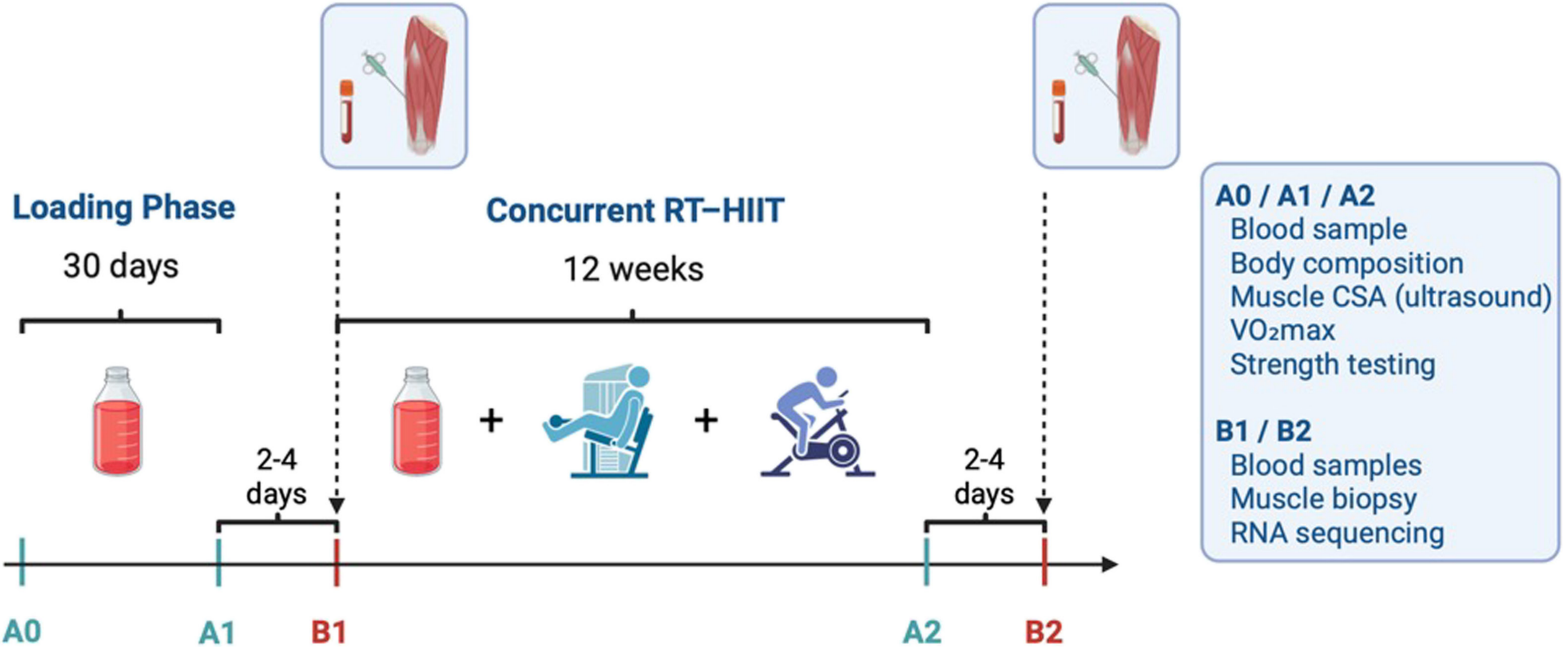
Study design and timeline.

Participants underwent baseline assessments (A0), including blood sampling, anthropometry, dual‐energy X‐ray absorptiometry (DXA), maximal oxygen uptake (V̇O_2max_) and strength testing. Following double‐blind randomization to either a polyphenol (PP) or placebo (PLA) group, participants completed a 30‐day supplementation loading phase without exercise. This was followed by a second assessment (A1) and a muscle biopsy (B1), performed prior to a 12‐week supervised concurrent training intervention consisting of RT (twice weekly) and HIIT (once weekly), with continued supplementation. Post‐intervention assessments (A2) and a second muscle biopsy (B2) enabled evaluation of physiological adaptations and skeletal muscle transcriptomic responses (Figure 1).

Details regarding the study design, participant characteristics, eligibility criteria and primary physiological outcomes have been reported previously [5]. The study was conducted in accordance with the Declaration of Helsinki and approved by the Regional Ethical Committee for Copenhagen (H‐22000157).

**FIGURE 2:**
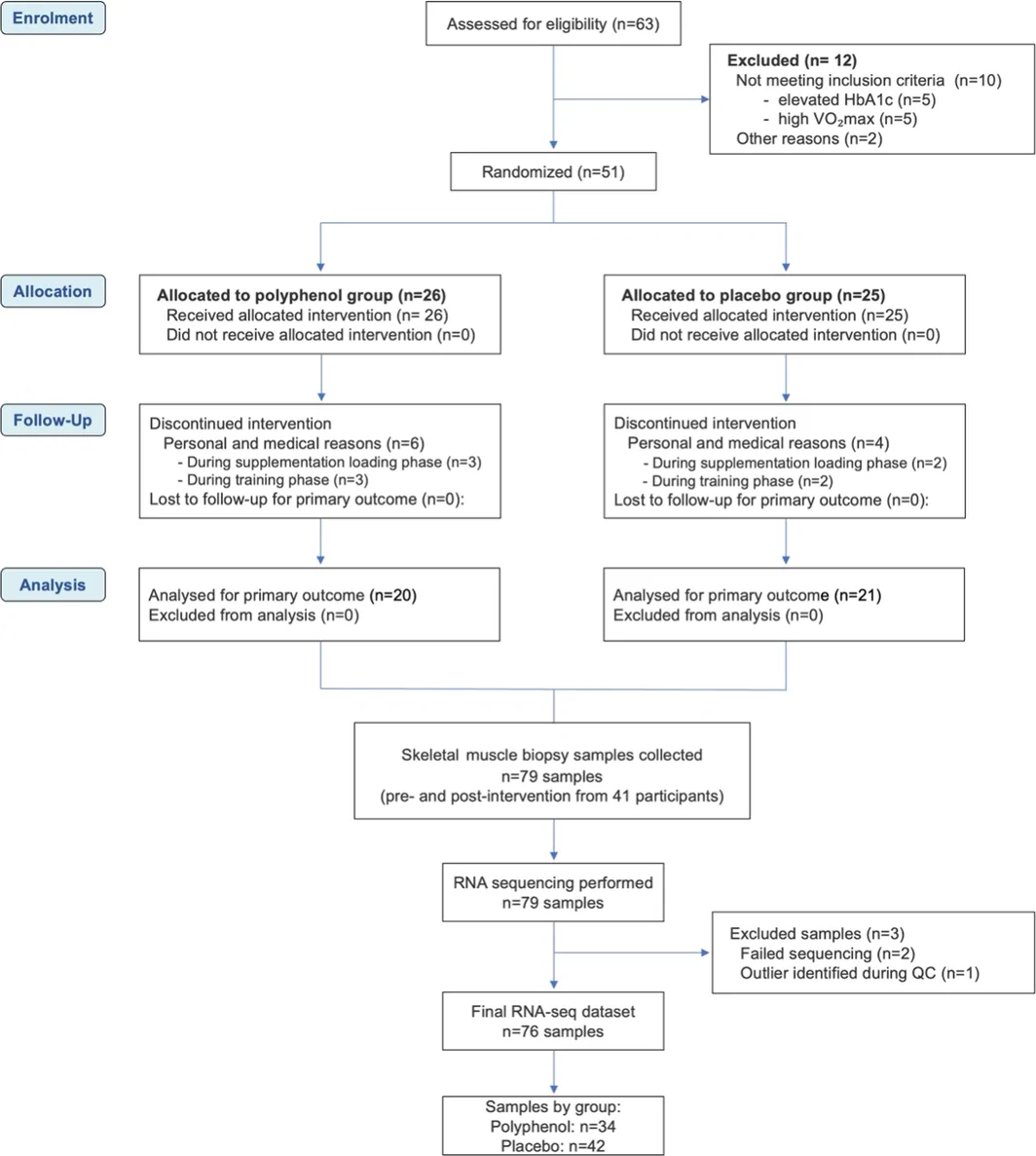
Participant flow and RNA sequencing data processing. Flow diagram illustrating participant recruitment, screening, allocation and retention throughout the study, as well as the processing of skeletal muscle samples for transcriptomic analysis. Of 162 individuals initially approached, 63 were eligible for screening and completed baseline assessments (A0). Following exclusion and dropout during the supplementation phase and training intervention, 41 participants completed the full protocol and underwent muscle biopsy sampling. A total of 79 muscle samples (pre‐ and post‐intervention) were obtained, of which 76 passed sequencing and quality control procedures and were included in the final transcriptomic analysis (polyphenol group: *n* = 34 samples; placebo group: *n* = 42 samples). Reasons for exclusion at each stage are detailed in the corresponding boxes.

Participant recruitment, allocation, retention and RNA sequencing sample processing are summarized in the CONSORT flow diagram (Figure 2). Overall, 41 adults (21 men and 20 women) aged 55–70 years were randomized into either a polyphenol (PP) supplementation group (mean age, 62 ± 4 years; range, 55–69) or a placebo (PLA) group (mean age, 61 ± 4 years; range, 55–70) in a double‐blind manner. Supplement allocation was managed by an independent researcher not involved in participant testing or data collection. Participants arrived in the morning after an overnight fast at the laboratory for baseline measurements (A0), which included venous blood sampling, body composition, maximal oxygen uptake (V̇O_2max_) and a battery of strength parameters. Following this initial visit, participants completed a 30‐day supplementation period (loading phase), during which they consumed either a polyphenol‐rich extract or a placebo once daily. The polyphenol supplement, extracted from red and blackcurrants, provided approximately 700 mg of polyphenols per dose. A full compositional profile of the polyphenol supplement, including mass spectrometry data, is available in the referenced study [5].

After the supplementation phase, participants returned for a second testing session (A1), conducted under identical conditions to baseline. Following a recovery period of 2–4 days, participants attended a third visit (B1) in the fasten state, during which blood samples and a skeletal muscle biopsy from the vastus lateralis were obtained.

Participants then undertook a 12‐week concurrent training programme, consisting of full‐body RT twice per week and an additional weekly session combining lower‐body RT with 7–9 min of HIIT, while continuing supplementation or placebo. Following the intervention, participants were tested again for the baseline characteristics (A2), and thereafter, participants returned for a final experimental visit (B2) that included blood sampling and muscle biopsy collection. For the present study, skeletal muscle samples collected before (B1, PRE) and after (B2, POST) the training intervention were used for RNA sequencing analyses.

### 2.2 Concurrent Training Intervention

The full description of the concurrent training programme has been published previously by Flensted‐Jensen et al. [5]. Briefly, participants completed a 12‐week supervised intervention consisting of two weekly RT sessions and one additional session combining RT with HIIT. RT sessions included multi‐joint and single‐joint exercises targeting major muscle groups (e.g., leg press, leg extension, leg curl, lat pulldown, seated row, chest press and shoulder press), with progressive overload from moderate to high intensities across the intervention period. Lower‐body exercises progressed from higher repetition ranges (≈12 RM) to lower repetition ranges (≈6 RM), whereas upper‐body exercises progressed from ~15 RM to ~10 RM. The combined RT + HIIT session incorporated lower‐body resistance exercises followed by short‐duration high‐intensity intervals performed on a cycle ergometer. HIIT intensity and volume were progressively increased throughout the intervention. All training sessions were supervised by qualified personnel to ensure proper technique, adherence and progression.

### 2.3 Muscle Biopsy Sampling

Muscle biopsy samples were collected from the *m. vastus lateralis* on days B1 (PRE) and B2 (POST), as previously described [5]. Snap‐frozen muscle biopsy samples with an average weight of 30 mg were freeze‐dried overnight (ScanVac, Denmark), dissected free of blood and connective tissue and subsequently stored at −80°C before transportation and further transcriptomics analysis.

### 2.4 RNA Extraction

To extract RNA from lyophilized muscle biopsies, a modified TRIzol reagent extraction protocol was used. Briefly, 500 μL of RNase‐free water (Sigma‐Aldrich, USA) and 7 μL of Proteinase K (Thermo Fisher Scientific, USA) were added to each sample and incubated at 50°C for 15 min. Subsequently, 1 mL of TRIzol reagent (Sigma‐Aldrich, USA) and 20 μL of 5 M acetic acid (Sigma‐Aldrich, USA) were added, and the sample was briefly vortexed. The suspension was transferred to a Lysing Matrix E tube (MP Biomedicals, USA) and homogenized for 40 s at 8000 rpm. Samples were then centrifuged for 10 min at 12 000 × g at 4°C, and the supernatant was transferred to a new 2.0‐mL LoBind tube (Eppendorf, Germany). After a 5‐min incubation at room temperature, 200 μL of chloroform (Fisher Chemical, USA) was added, vortexed for 15 s and incubated again at room temperature for 10 min. Samples were centrifuged for 15 min at 12 000 × g at 4°C, and the upper aqueous phase was transferred to a new 2.0‐mL LoBind tube. Next, 500 μL of isopropyl alcohol (Fisher Chemical, USA) was added and gently mixed. Following a 10‐min incubation at room temperature, samples were centrifuged for 10 min at 12 000 × g at 4°C. The supernatant was removed carefully without disturbing the RNA pellet. Subsequently, 1 mL of 75% ethanol (Kalsnavas elevators Ltd, Latvia) was added, briefly vortexed and centrifuged for 5 min at 7500 × g at 4°C. Ethanol was then removed, and samples were left to air dry for 5–10 min, ensuring the pellet did not dry completely. Finally, 30 μL of RNase‐free water (Sigma‐Aldrich, USA) was added, and samples were incubated in a shaking rotator at 55°C for 10 min at 600 rpm to facilitate RNA elution. RNA concentration was measured using the Qubit RNA HS Assay Kit and a Qubit 4.0 fluorometer (both from Thermo Fisher Scientific, USA). RNA quality was assessed using the 4200 TapeStation system with High Sensitivity RNA ScreenTape, Sample Buffer and Ladder (Agilent Technologies, USA).

### 2.5 RNA Sequencing

Extracted RNA was subsequently used for sequencing at CeGaT GmbH (Tübingen, Germany). Briefly, for each muscle sample, 10 ng of total RNA was used as input for library preparation using the SMART‐Seq Stranded kit (Takara Bio, Japan). All successful libraries were sequenced on an Illumina NovaSeq 6000 platform using 2 × 101 bp paired‐end reads (Illumina, USA).

Sequence data analysis was outsourced to CeGaT GmbH. In brief, raw read demultiplexing was performed using Illumina bcl2fastq (version 2.20). Adapter trimming was conducted with Skewer (version 0.2.2) [9], and read quality was assessed using FastQC (version 0.11.5‐cegat) [10]. Trimmed reads were aligned to the human reference genome GRCh38 using STAR (version 2.7.3) [11]. Gene‐level counts were then generated by merging aligned data into a single table based on gene IDs, using the pandas library in the Python v.3.13.7 programming environment. Raw FASTQ files and the associated count tables have been deposited in RSU Dataverse.

### 2.6 In Silico Estimation of Fibre Type Composition

The proportion of slow‐ and fast‐twitch skeletal muscle fibre types was estimated from the raw gene‐level count dataset using the FibeRtypeR web application (https://muscleapps.ugent.be, accessed 23 January 2026) [12]. Based on a validated gene‐signature matrix specific to slow‐twitch (Type I) and fast‐twitch (Type II) fibres, relative fibre‐type composition was inferred for each RNA‐sequenced sample and included as a covariate in subsequent analyses. Although immunohistochemical fibre‐type measurements were available for a subset of samples from this cohort [5], these data were incomplete for the RNA‐sequencing dataset. FibeRtypeR estimates were therefore used to enable consistent adjustment for fibre‐type composition across all transcriptomic samples without additional sample exclusion.

### 2.7 Single Gene Differential Expression and Pathway‐Level Information Analysis

Differential gene expression analysis was performed using the edgeR package (version 4.7.3) [13]. Raw sequence counts were first transformed into counts per million (CPM), and genes were retained if they had a CPM ≥ 0.5 in at least two samples. Data normalization was subsequently performed using the trimmed mean of *M*‐values (TMM) method. To identify potential outliers, *z*‐scores were calculated on the first principal coordinate, and samples with |*z*| > 3 were considered outliers and removed. To assess potential confounding effects, sex, age and fibre composition were tested as covariates using Genewise Likelihood Ratio Tests implemented in edgeR. Sex was identified as a strong confounder, affecting 1040 genes, whereas fibre composition affected 633 genes. Age had a weaker effect, influencing only nine genes. Therefore, sex, age and fibre composition were included as covariates in all downstream models; for models containing paired samples, individual and fibre composition were included as covariates.

Differential expression was assessed using a Benjamini–Hochberg‐adjusted false discovery rate (FDR) threshold of ≤ 0.05. An additional absolute log_2_ fold‐change (log_2_FC) threshold of > 1 was applied to prioritize transcripts showing both statistical significance and a comparatively larger estimated magnitude of change. Throughout the manuscript, transcripts reaching FDR ≤ 0.05 are described as FDR‐significant, whereas those satisfying both FDR ≤ 0.05 and |log_2_FC| > 1 are described as meeting the combined statistical and effect‐size criterion. Complete results for all FDR‐significant transcripts from the main differential‐expression analyses are provided in Table S1. Group‐based comparisons were conducted between the PP and PLA groups at the baseline (e.g., PRE), as well as between PRE and POST samples irrespective to the group. In addition, an interaction model was constructed to test whether the change from pre‐ to post‐training intervention in the PP group significantly differed from the corresponding change in the PLA group. Heatmaps were generated using ComplexHeatmap (version 2.25.2) [14], based on the top 200 transcripts with the lowest FDR‐adjusted *p*‐values from each tested model.

Gene annotations were retrieved using the biomaRt package (version 2.65.0) [15]. Visualization of differential expression results was carried out using ggplot2 (version 3.5.2), including volcano plots and individual expression profiles for significant genes. Gene ontology (GO) enrichment analysis was conducted using clusterProfiler (version 4.17.0) [16] and org.Hs.eg.db (version 3.21.0) [17], whereas Reactome pathway analysis was performed using ReactomePA (version 1.53.0) [18]. KEGG pathway enrichment was also carried out using clusterProfiler.

Gene set enrichment analysis (GSEA) was performed using the fgsea version 1.36.2 package [19]. Genes were ranked using the likelihood‐ratio (LR) statistic, with the direction of the ranking determined by the sign of the log_2_ fold change (ranking statistic = sign (log_2_FC) × LR). Only genes with valid gene symbols were retained, and duplicate symbols were removed by keeping the first occurrence. Hallmark gene sets for *Homo sapiens* were obtained from the MSigDB database using msigdbr version 25.1.1 [20]. Enrichment analysis was performed using gene sets containing 10–500 genes. Statistical significance was determined using BH *p*‐value adjustment. Top enriched pathways were visualized using ggplot2.

### 2.8 Statistical Analyses

For most outcome measures, the main effect of time was examined using mixed modelling, using absolute values for the dependent variable and time points as repeated measures/observations with random intercepts for each subject. In general, for continuous variables, the effects of polyphenol supplementation (compared with PLA) were investigated using linear mixed‐effects models with the relative change from baseline being defined as the dependent variable and the supplementation arm being defined as the fixed effect. All data are presented as mean ± standard deviation or estimated mean difference (EMD), as well as the 95% confidence interval, unless stated otherwise.

## 3 Results

### 3.1 Participant Characteristics

A total of 79 skeletal muscle biopsies obtained before and after the intervention were subjected to RNA sequencing, of which 76 passed sequencing and quality control procedures. Two samples failed sequencing, and one pre‐intervention sample was excluded as an outlier following quality assessment. Therefore, the final RNA‐seq dataset comprised samples from 38 participants: 17 in the PP group and 21 in the PLA group (Table S2).

Baseline characteristics of participants included in the transcriptomic analyses are presented in Table 1 and were comparable between groups. A detailed overview of sample inclusion, exclusion and RNA sequencing sample processing is provided in Figure 2.

**TABLE 1:** Baseline characteristics of study participants.

	*n*	Placebo (PLA)	*n*	Polyphenol (PP)	*p*
		Mean ± SD		Mean ± SD	
Female (%)	10	47.6	9	52.9	
Male (%)	11	52.4	8	47.1	
Age (years)	21	61.8 ± 4.2	17	60.7 ± 4.1	0.418
BMI (kg·m^−2^)	21	26.1 ± 3.4	17	26.0 ± 2.2	0.897
Fat mass (kg)	21	26.4 ± 7.8	17	27.3 ± 6.4	0.704
Total lean mass (kg)	21	52.6 ± 10.3	17	50.9 ± 8.2	0.592
VL CSA (cm^2^)	21	17.1 ± 6.6	15	13.7 ± 6.4	0.139
MVC (Nm)	20	171.8 ± 49.5	17	168.9 ± 57.6	0.870
Muscle power (W)	21	198.0 ± 71.7	17	184.8 ± 58.6	0.545
VO_2max_ (mL·kg^−1^·min^−1^)	20	28.9 ± 4.5	17	29.3 ± 4.8	0.928

Abbreviations: BMI: body mass index; CSA: cross‐sectional area; MVC: maximal voluntary contraction; VL: vastus lateralis; VO_2max_: maximal oxygen uptake volume.

### 3.2 Baseline Transcriptomic Differences Between Placebo and Polyphenol Groups

**FIGURE 3:**
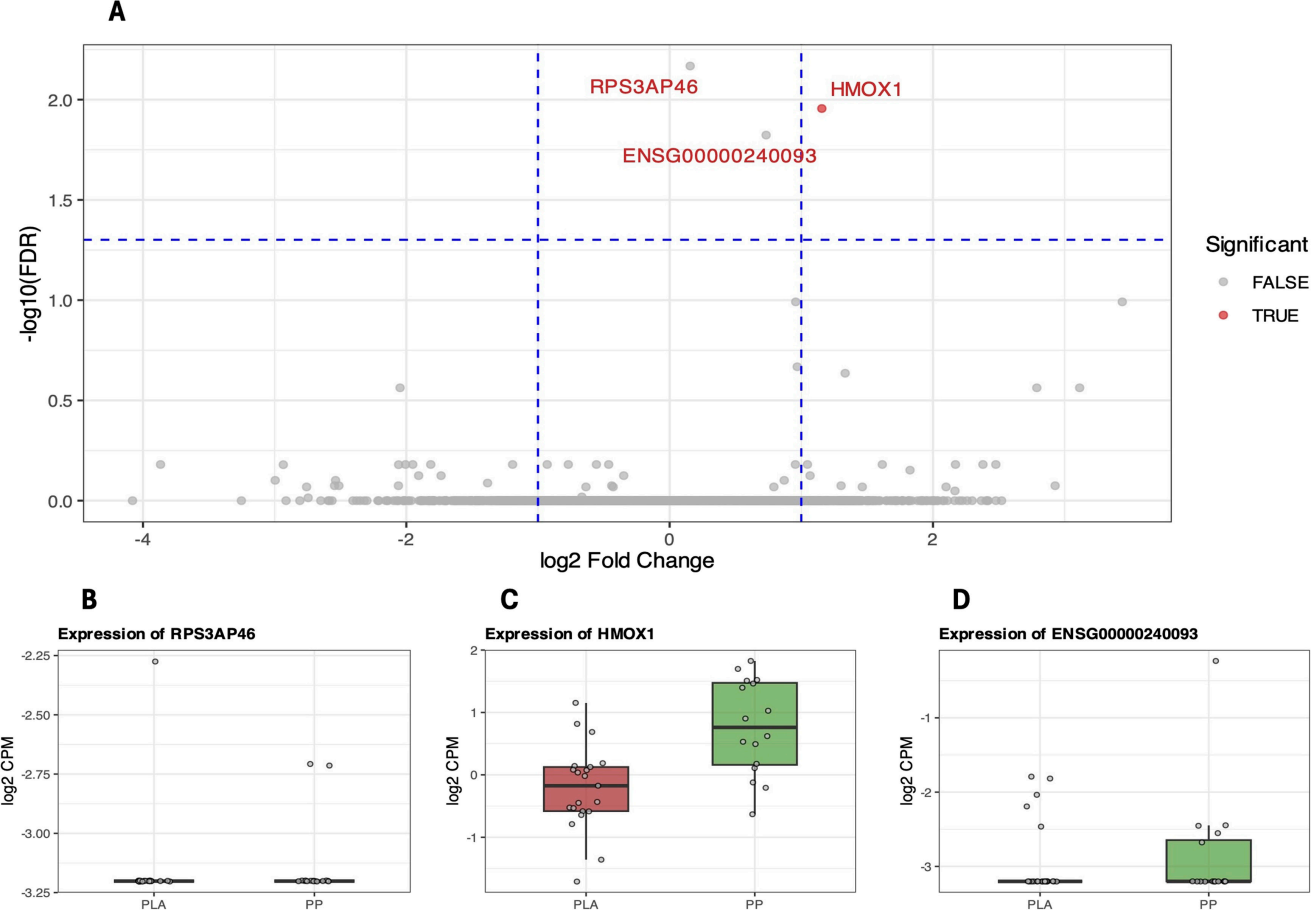
Baseline differential expression of skeletal muscle transcriptomes between the polyphenol and placebo groups before the training intervention (PP‐PRE vs PLA‐PRE). Panel A: Volcano plot. The *x*‐axis shows log_2_ fold change, and the *y*‐axis shows −log_10_(FDR‐adjusted *p*‐value). Transcripts meeting the combined statistical and effect‐size criterion (FDR ≤ 0.05 and |log_2_FC| > 1) are highlighted in red, and selected transcripts are labelled. Panels B–D: Individual expression values for FDR‐significant transcripts in the polyphenol (PP) and placebo (PLA) groups.

Comparison of skeletal muscle transcriptomes between the PLA and PP groups at baseline (PLA‐PRE vs. PP‐PRE) is shown in Figure 3A. Three transcripts reached FDR significance (≤ 0.05) after adjustment for age, sex and muscle fibre composition: *RPS3AP46* (FDR = 0.007; log_2_FC = 0.16; Figure 3B), *HMOX1* (FDR = 0.011; log_2_FC = 1.16; Figure 3C) and *ENSG00000240093* (FDR = 0.015; log_2_FC = 0.73; Figure 3D). The magnitude of differential expression was modest. Although all three transcripts reached FDR significance, only *HMOX1* additionally exceeded |log_2_FC| > 1 threshold, indicating minimal transcriptomic differences between groups at baseline (Table S1). *HMOX1*, a gene involved in oxidative stress response, showed a small upregulation in the PP group; however, the modest estimated effect size suggests no biologically meaningful separation between groups prior to the intervention. Although some gene sets showed nominal enrichment, these signals were modest and lacked a coherent biological pattern, supporting the absence of meaningful baseline transcriptomic differences between PP and PLA groups.

### 3.3 Effects of Polyphenol Supplementation on Transcriptomic Responses to Training

**FIGURE 4:**
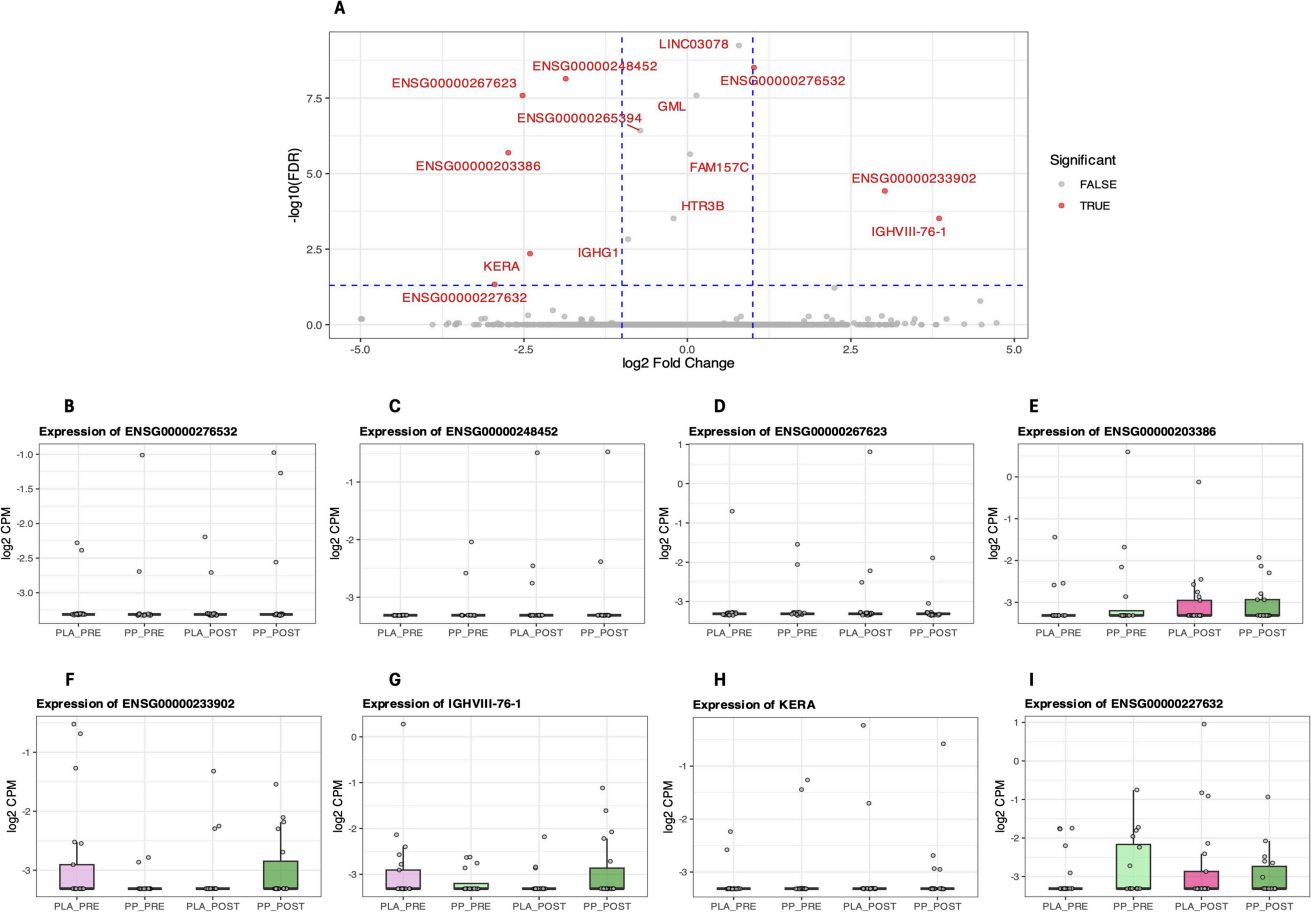
Integrated analysis of transcriptomic responses to training with polyphenol supplementation. Panel A: Volcano plot showing differential gene expression for the interaction contrast ((POST–PRE in PP) − (POST–PRE in PLA)). The *x*‐axis represents log_2_ fold change, and the *y*‐axis shows −log_10_(FDR‐adjusted *p*‐value). Red dots indicate transcripts meeting the combined statistical and effect‐size criterion (FDR ≤ 0.05 and |log_2_FC| > 1). Panels B–I: Individual expression values for transcripts meeting both significance thresholds (FDR < 0.05 and |log_2_FC| > 1) in the PP and PLA groups at the two time points.

To determine whether polyphenol supplementation modulated the transcriptional response to the training intervention, interaction analyses were performed comparing changes over time between the PP and PLA groups, adjusting for age, sex and muscle fibre composition. The group‐by‐time interaction analysis identified 14 FDR‐significant transcripts, of which eight additionally exceeded |log_2_FC| > 1 (Figure 4 and Table S1). Transcripts meeting the combined statistical and effect‐size criterion included *KERA* and *IGHVIII‐76‐1*, along with poorly annotated transcripts. As shown in the volcano plot (Figure 4A), most transcripts clustered around a log_2_ fold change close to zero, indicating that the between‐group differences in training‐induced expression changes were generally small. Only a limited subset of transcripts showed both statistical significance and larger effect sizes. However, inspection of their individual expression profiles revealed low or sparse expression and marked inter‐individual variability, reducing confidence that these signals represented a coherent supplementation‐related transcriptional response (Figures 4B–I and S1). Consistent with this, inspection of individual expression profiles revealed low or sparse expression and marked inter‐individual variability for several of these transcripts, rather than consistent within‐subject changes over time. At the global level, the heatmap of top‐ranked genes (Figure S2) did not reveal clear clustering by group or timepoint, further supporting the absence of a robust transcriptional signature associated with polyphenol supplementation. Functional enrichment analyses also lacked biological coherence, with GO and Reactome results largely driven by a single gene (*KERA*), and no significantly enriched pathways identified in KEGG (Figure S3). Collectively, these findings indicate that polyphenol supplementation did not meaningfully modulate the skeletal muscle transcriptomic response to training.

### 3.4 Pooled Effects of the Training Intervention on the Skeletal Muscle Transcriptome

**FIGURE 5:**
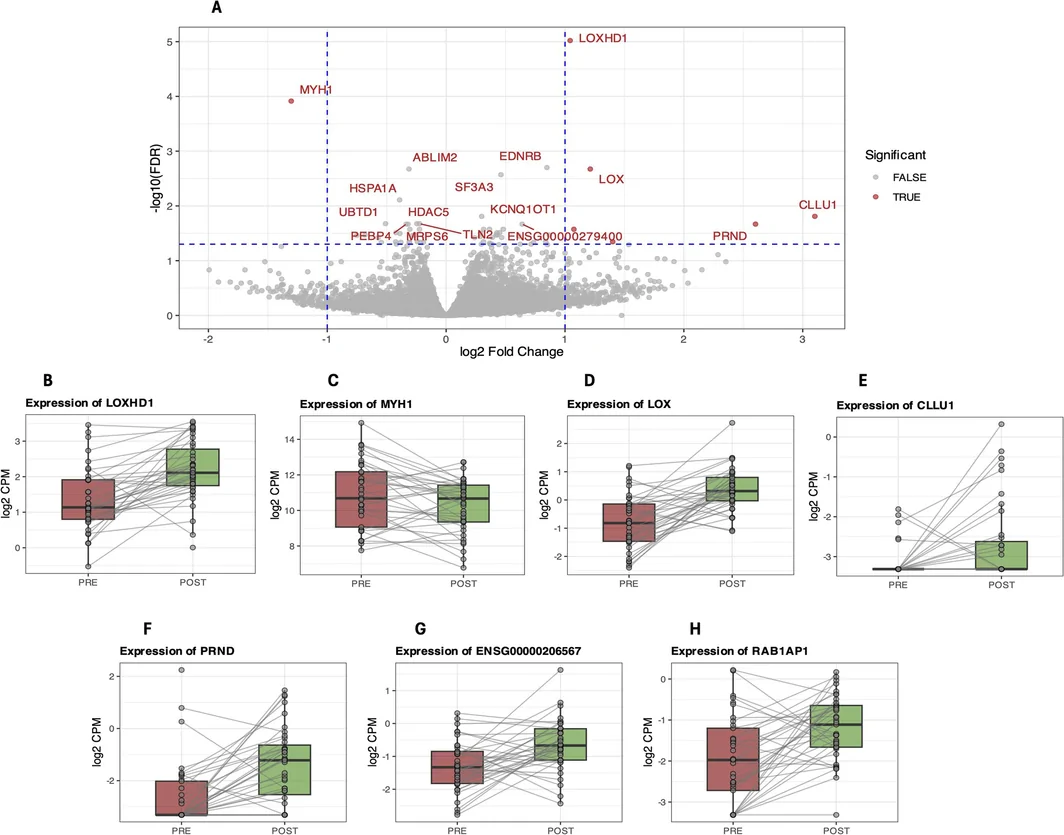
Pooled transcriptomic response to the training intervention. Panel A: Volcano plot showing differential gene expression between PRE and POST intervention across all participants. Each point represents a transcript, plotted according to log_2_ fold change and −log_10_(FDR). Red dots indicate transcripts meeting the combined statistical and effect‐size criterion (FDR ≤ 0.05 and |log_2_FC| > 1). Panels B–H: Individual expression values for transcripts meeting both significance thresholds (FDR < 0.05 and |log_2_FC| > 1) at the two time points. PRE and POST measurements from the same individual are connected by lines.

Differential expression analysis comparing PRE and POST samples using a paired model adjusted for participant and muscle fibre composition identified 60 FDR‐significant transcripts, of which seven additionally met the combined statistical and effect‐size criterion (FDR ≤ 0.05 and |log_2_FC| > 1) (Figure 5, Table S1 and Figure S4). The volcano plot (Figure 5A) showed that most transcripts clustered around relatively small log_2_ fold changes, indicating that the training‐associated transcriptional response was broader than suggested by the combined criterion alone, but predominantly modest in magnitude.

Among the seven transcripts meeting the combined statistical and effect‐size criterion, six were upregulated, and one was downregulated following training. *CLLU1* (log_2_FC = 3.10; FDR = 0.0155) and *PRND* (log_2_FC = 2.60; FDR = 0.02) showed the largest positive changes, whereas *MYH1* showed the most pronounced downregulation (log_2_FC = −1.30; FDR = 0.0001) (Figure 5E,F,C, respectively). The remaining FDR‐significant transcripts generally showed smaller estimated effect sizes (|log_2_FC| ≤ 1), consistent with a predominantly low‐magnitude transcriptional response (Table S1 and Figure S4). Gene expression changes were relatively consistent within individuals, and heatmap analysis indicated a subtle overall shift without clear clustering (Figure S5). No robust pathway enrichment was detected. Selected transcript‐level changes included genes related to extracellular matrix biology (e.g., *LOX*) and contractile phenotype (e.g., *MYH1*) (Figure S6).

Additional interaction analyses were performed to determine whether sex‐specific responses may have been masked by the pooled analysis. No transcripts showed a significant sex‐by‐time interaction after correction for multiple testing (FDR < 0.05). Similarly, no significant sex‐by‐group‐by‐time interactions were detected, providing no statistically significant evidence of sex‐specific transcriptional responses to either training or polyphenol supplementation in the present dataset.

## 4 Discussion

The present study provides a transcriptome‐wide characterization of skeletal muscle adaptations to a 12‐week concurrent resistance and high‐intensity interval training (RT–HIIT) intervention in middle‐aged and older adults, with and without polyphenol supplementation. The main findings were (i) the overall transcriptional response to training was statistically detectable but predominantly modest in magnitude and (ii) polyphenol supplementation did not meaningfully modulate the transcriptomic response.

### 4.1 Modest Transcriptomic Remodelling Following Concurrent Training

A central observation of this study is that the training‐associated transcriptomic response was broader than suggested by the combined statistical and effect‐size criterion alone, but predominantly modest in magnitude. In the pooled PRE–POST analysis, 60 transcripts reached FDR significance, whereas only seven additionally exceeded |log_2_FC| > 1. The additional |log_2_FC| > 1 threshold used to prioritize transcripts with larger estimated effect sizes may have reduced sensitivity to more modest but potentially biologically relevant transcriptional changes. Thus, in the context of the 60 FDR‐significant transcripts identified in the pooled analysis, the limited number of transcripts additionally exceeding the fold‐change threshold should not be interpreted as evidence of an absence of transcriptional adaptation. This finding contrasts with the robust physiological adaptations previously reported in this cohort, including improvements in muscle mass, strength, aerobic capacity and mitochondrial function [5, 6]. Accumulating evidence indicates that chronic exercise‐induced transcriptomic adaptations in human skeletal muscle are often subtle and distributed across multiple genes with small effect sizes rather than large‐scale transcriptional shifts. Notably, recent single‐nucleus RNA sequencing data have similarly demonstrated a limited number of DEGs following high‐intensity training, even when using more sensitive cell‐specific approaches [21, 22, 23].

Large‐scale multi‐omic analyses have further demonstrated that most exercise‐induced molecular adaptations fall within a relatively narrow fold‐change range, reflecting coordinated fine‐tuning of biological systems [21]. Thus, the predominance of modest transcriptional effect sizes observed in the present study is consistent with previous reports of relatively subtle transcriptional adaptations to chronic exercise; however, methodological factors, inter‐individual variability and limited statistical power may also have contributed to the number and magnitude of detected differential‐expression signals.

### 4.2 Extracellular Matrix Remodelling

Among the upregulated transcripts, *LOX* (encoding lysyl oxidase) represents a biologically plausible signal in the context of extracellular matrix remodelling. *LOX* is a key enzyme involved in collagen and elastin cross‐linking and plays a central role in ECM remodelling and tissue mechanical properties. Exercise‐induced ECM remodelling has emerged as a consistent molecular response to both resistance and endurance training, facilitating force transmission and structural integrity within skeletal muscle [24, 25]. Previous studies have reported coordinated upregulation of ECM‐related genes, including collagen isoforms, matrix remodelling enzymes and adhesion molecules, following exercise interventions [26]. In this context, the observed upregulation of LOX is consistent with an extracellular matrix‐related transcriptional response to training. This finding is of particular interest given recent observations from the same cohort showing a prominent localization of advanced glycation end‐products within the skeletal muscle extracellular matrix, although their abundance was not altered by the 12‐week training intervention [27]. Nevertheless, in the absence of robust pathway‐level enrichment in the present transcriptomic analysis, the LOX response should be interpreted as an individual transcript‐level signal rather than evidence of coordinated extracellular matrix remodelling.

### 4.3 MYH1 Downregulation and Contractile Phenotype Adaptation

A notable finding was the downregulation of *MYH1*, encoding the myosin heavy chain IIx isoform. *MYH* isoforms are key determinants of muscle fibre contractile properties, with *MYH1* associated with fast glycolytic fibres (Type IIx). Exercise interventions combining resistance and endurance components have been shown to induce shifts towards a more oxidative phenotype, often characterized by transitions from Type IIx towards Type IIa fibres [28]. Interestingly, histological analyses previously performed in the same cohort showed that training increased overall Type II fibre cross‐sectional area, whereas Type I fibre size and the relative distribution of Type I and Type II fibres remained unchanged [5]. However, because the histological analysis did not separately quantify Type IIa and IIx fibres, the present findings do not allow us to determine whether the reduction in MYH1 reflects a shift between fast‐fibre subtypes. Accordingly, the MYH1 response is best interpreted as a transcript‐level change related to the fast contractile phenotype rather than direct evidence of fibre‐type transition.

### 4.4 Polyphenol‐Related Transcriptomic Responses

The group‐by‐time interaction analysis identified statistically significant transcript‐level differences between the PP and PLA groups, indicating that the temporal expression profiles of a limited set of transcripts differed between supplementation conditions. Among these signals, *KERA* showed one of the larger estimated interaction effects and met both the FDR and fold‐change criteria. *KERA* encodes keratocan, a keratan sulphate proteoglycan localized to the extracellular matrix, providing a biologically interpretable link to matrix‐related processes. However, *KERA* was expressed at a relatively low level in the present dataset (logCPM = −2.91), and the interaction signal did not occur alongside a consistent transcriptional response across other extracellular matrix‐related genes. Accordingly, the isolated *KERA* response should not be interpreted as evidence of coordinated extracellular matrix remodelling attributable to polyphenol supplementation. Several other FDR‐significant interaction transcripts showed low or sparse expression, substantial inter‐individual variability or limited functional annotation, and the interaction effects did not converge on a coherent pathway‐level signature. Thus, although the interaction analysis provides statistical evidence that supplementation was associated with differential temporal expression of specific transcripts, the present data do not support a robust coordinated modification of the skeletal muscle transcriptional response to training by polyphenol supplementation. This interpretation is further supported by complementary findings from the same cohort. Polyphenol supplementation did not augment training‐induced improvements in skeletal muscle mitochondrial bioenergetics or redox balance, did not exert a clear additional effect on the inflammatory response to training and did not alter skeletal muscle AGE accumulation [5, 6]. Although selected supplementation‐related differences were observed for mitochondrial respiratory capacity and circulating antioxidant responses, their interpretation was uncertain and they did not translate into enhanced physiological adaptations. Thus, the absence of a coherent supplementation‐related transcriptomic signature in the present study is consistent with the overall pattern observed across complementary molecular and physiological outcomes from the same trial.

### 4.5 Biological Interpretation of the Modest Transcriptomic Response

The absence of robust pathway‐level enrichment limits the extent to which the observed transcript‐level changes can be interpreted as coordinated biological adaptations. Nevertheless, the FDR‐significant transcriptional response indicates that training was associated with detectable changes in skeletal muscle gene expression, although these were predominantly modest in magnitude and did not converge on a coherent pathway‐level signature. Importantly, transcriptomic analyses do not capture key adaptive processes such as ribosome biogenesis, which plays a central role in regulating translational capacity and muscle hypertrophy [29]. Given that ribosomal RNA constitutes most of the total RNA in skeletal muscle and is typically excluded from mRNA sequencing approaches, relevant adaptations at the translational level may not be detected using transcriptomic methods alone. From a clinical perspective, preservation of physical function is particularly relevant in the context of frailty, a multidimensional syndrome that frequently coexists with cardiometabolic and cognitive impairment in older adults [30, 31, 32]. Shared pathophysiological features, including chronic inflammation, oxidative stress, mitochondrial dysfunction and reduced physiological reserve, may contribute to the association between frailty and cardiometabolic disease [30]. In addition, reported associations between impaired physical performance, cardiovascular abnormalities and cognitive dysfunction in frail populations further illustrate the multisystem nature of vulnerability in later life [31, 32]. These observations reinforce the clinical importance of maintaining physical function during ageing, whereas frailty and cognitive outcomes were not directly evaluated in the present study.

### 4.6 Strengths and Limitations

This study has several strengths, including its randomized, double‐blind design, the integration of exercise and nutritional interventions, the use of paired transcriptomic analyses and the adjustment for key confounders such as sex, age and muscle fibre composition.

A key strength of the present study is the adjustment for skeletal muscle fibre type composition, which emerged as a major determinant of gene expression variability. Indeed, fibre type composition influenced the expression of substantially more genes than age, highlighting its dominant role in shaping the skeletal muscle transcriptome. This observation is consistent with previous studies demonstrating that slow‐ and fast‐twitch fibres exhibit markedly distinct transcriptional profiles, which strongly influence bulk RNA sequencing results [33]. Importantly, many transcriptomic studies in human skeletal muscle have not accounted for fibre type composition, potentially confounding the interpretation of exercise‐induced molecular adaptations. Failure to adjust for this factor may result in apparent differences in gene expression that reflect baseline phenotypic heterogeneity rather than true intervention effects. By incorporating in silico–estimated fibre type proportions as a covariate, the present study reduces the likelihood of detecting spurious differential expression driven by underlying muscle composition. Although this adjustment may have reduced the number of transcripts reaching statistical significance, it enhances the specificity and physiological interpretability of the findings. Therefore, the modest transcriptomic response reported here should be interpreted within a more stringent and biologically informed analytical framework.

However, several limitations should be acknowledged. First, the sample size may limit the statistical power to detect small but biologically relevant changes. Second, the use of bulk RNA sequencing precludes the identification of cell‐type–specific adaptations. Third, transcriptomic analyses do not capture post‐transcriptional or translational regulations. Finally, RNA quality was relatively low, and substantial RNA degradation may have reduced transcript detection sensitivity and increased variability in gene expression estimates. Further improvements in sample handling, including the introduction of RNA‐stabilizing reagents such as RNALater, may help improve RNA quality for the downstream applications [34]. Nevertheless, the use of a library preparation protocol compatible with degraded RNA, together with short‐read sequencing, enabled successful library generation and sequencing across samples, although degradation‐related biases cannot be excluded. In addition, although the 10‐ng RNA input was within the recommended range for the SMART‐Seq Stranded library preparation protocol, the relatively low absolute RNA input may have reduced library complexity and sensitivity for detecting very low‐abundance transcripts and increased variability in their quantification. Importantly, the adjustment for muscle fibre type composition may have further contributed to the reduced number of detected DEGs. Given the marked transcriptional differences between slow‐ and fast‐twitch fibres, failure to account for fibre type heterogeneity may inflate the number of apparent DEGs in bulk RNA sequencing analyses. Collectively, sample size, inter‐individual variability, RNA integrity, low absolute RNA input and analytical adjustment for fibre‐type composition may all have influenced the number and magnitude of detected differential‐expression signals. These factors should therefore be considered when interpreting the extent of the observed transcriptomic response.

## 5 Conclusion

Overall, these findings indicate that concurrent RT–HIIT induces a predominantly low‐magnitude transcriptional response in ageing skeletal muscle. Selected transcript‐level changes involved genes related to extracellular matrix biology and contractile phenotype without robust pathway‐level enrichment. Polyphenol supplementation did not meaningfully modify the skeletal muscle transcriptional response to training.

## Funding

This work was supported by the ‘Systemic and muscle tissue–specific metabolic adaptations to EXercise and POlyphenol supplementation in ageing: modulatory effects of SEstrins (EXPOSE)’ (01.04.2024.‐31.03.2026); ‘RSU internal and RSU with LSPA external consolidation’ (No. 5.2.1.1.i.0/2/24/I/CFLA/005 and Grant No. RSU/LSPA‐PA‐2024/1‐0007); and grant from the Center for Healthy Aging and from Asiros Nordic (Sorø, Denmark).

## Conflicts of Interest

The authors declare no conflicts of interest.

## Supporting information


**Figure S1:** Representation of DEGs as a response to training with polyphenol supplementation.
**Figure S2:** Integrated analysis of transcriptomic responses to training with polyphenol supplementation.
**Figure S3:** Gene set enrichment analysis (GSEA) of baseline skeletal muscle transcriptomic differences between polyphenol and placebo groups.
**Figure S4:** Effects of training intervention on the skeletal muscle transcriptome.
**Figure S5:** Integrated analysis of transcriptomic responses in All‐PRE and All‐POST samples.
**Figure S6:** Gene set enrichment analysis (GSEA) of the pooled PRE–POST skeletal muscle transcriptomic response.

**Table S1:** FDR‐significant transcripts in the main differential‐expression analyses.
**Table S2:** Sample metadata, RNA quality‐control metrics, sequencing characteristics and mapping statistics.

## Data Availability

Data are available on RSU Dataverse: DOI 10.48510/FK2/O5DUSW.
