# Supplementary material for: Skeletal Muscle Transcriptomic Responses to Concurrent Training With Polyphenols in Ageing Adults: A Randomized Trial
Source: J Cachexia Sarcopenia Muscle. 2026 Sep 25;17(5):e70391. doi: 10.1002/jcsm.70391 (PMC13613424; doi:10.1002/jcsm.70391)

Supplementary Figure S1. Representation of DEGs as a response to training with polyphenol supplementation.

Expression profiles of differentially expressed genes (DEGs), that were significant at FDR < 0.05 but did not meet the $|log_2FC| > 1$ threshold. Boxplots with individual data points show normalized expression (log_2 counts per million, CPM) across groups and time points. Most genes exhibit low expression levels, high inter-individual variability, and lack of consistent within-subject changes.

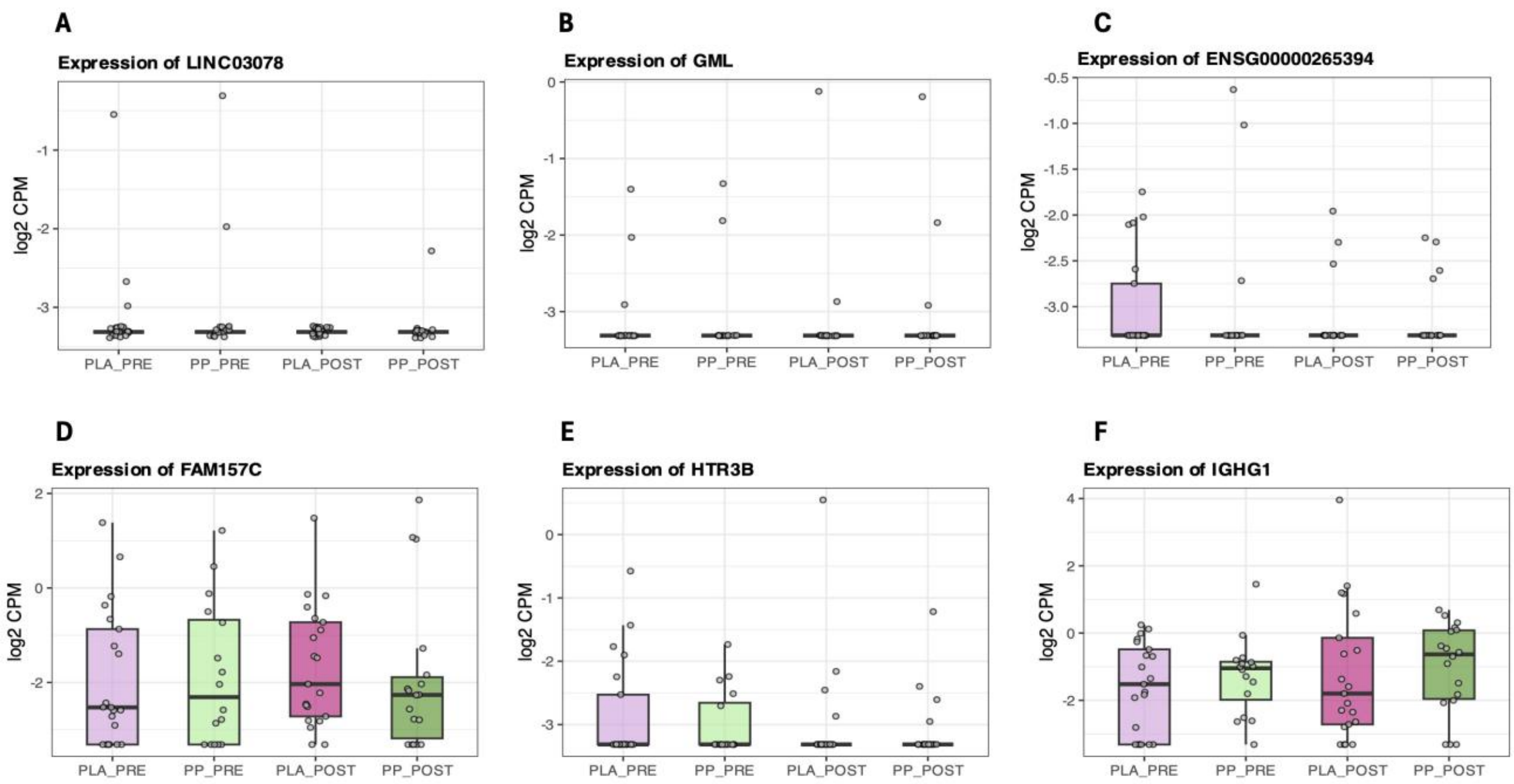

Supplementary Figure S2. Integrated analysis of transcriptomic responses to training with polyphenol supplementation.

Heatmap of the top 200 ranked genes (by FDR) from the interaction analysis. Expression values are scaled by gene (Z-score). Rows represent genes and columns represent individual samples across pre- and post-intervention time points. No clear clustering by group or time point is observed, indicating the absence of a coherent transcriptomic response to polyphenol supplementation

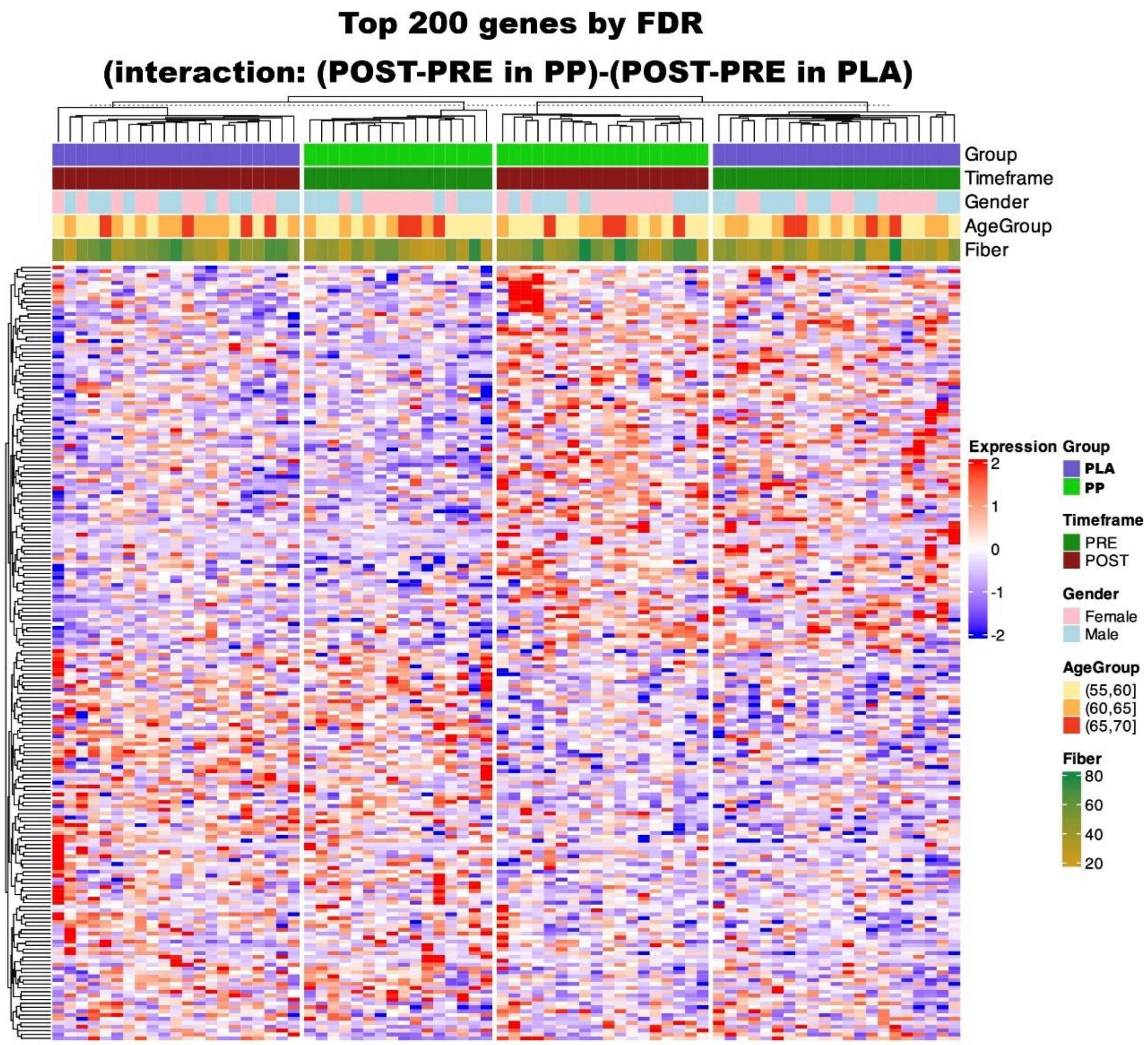

Supplementary Figure S3. Gene set enrichment analysis (GSEA) of baseline skeletal muscle transcriptomic differences between polyphenol and placebo groups.

(A) Enrichment plots for selected Hallmark pathways identified by FGSEA, showing the distribution of pathway genes across the ranked transcriptome. (B) Bubble plot of normalized enrichment scores (NES) for Hallmark gene sets. Dot size reflects gene set size and colour indicates FDR-adjusted p-value. Positive NES values indicate pathways enriched among transcripts with higher expression in the polyphenol group at baseline.

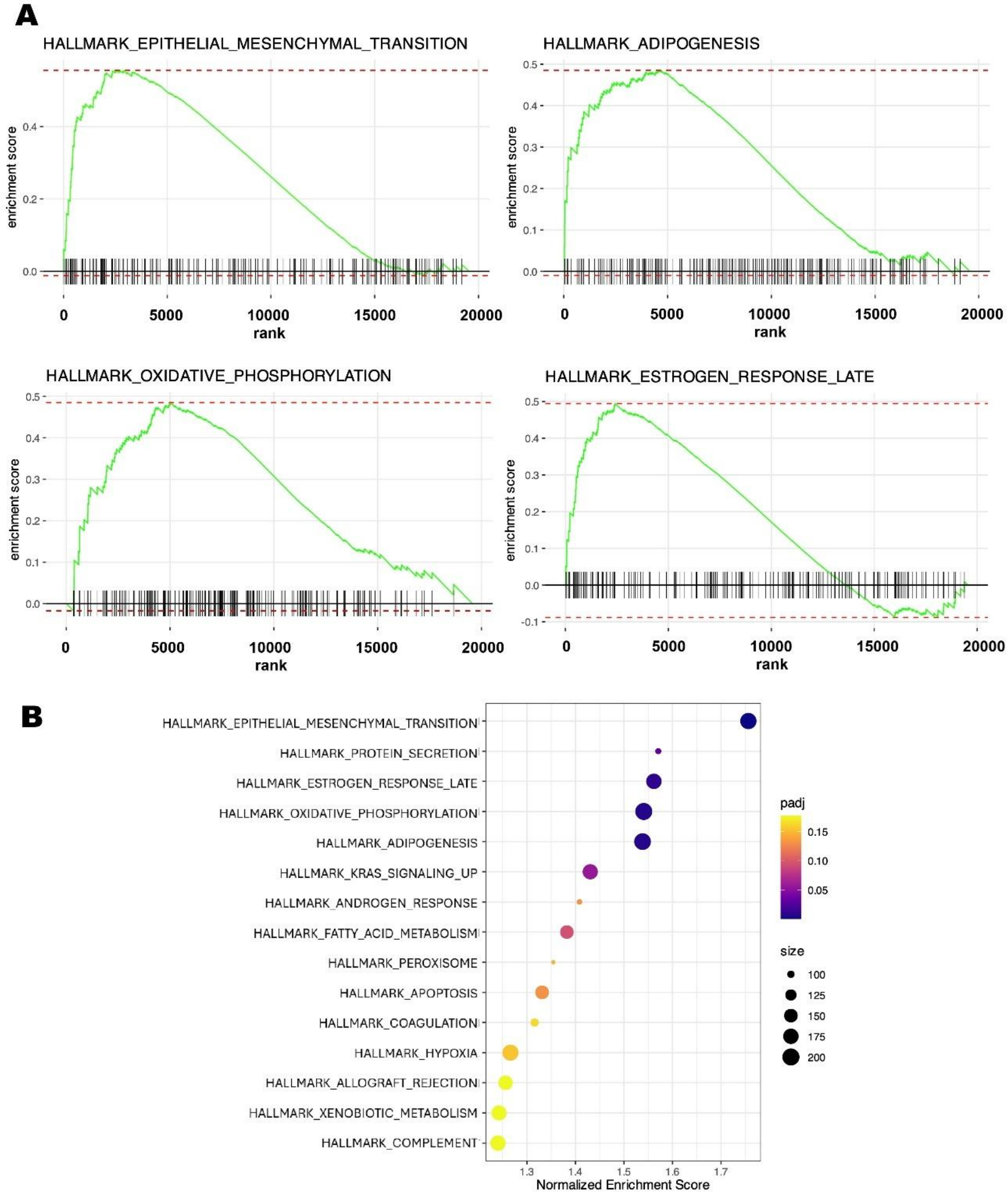

Supplementary Figure S4. Effects of training intervention on the skeletal muscle transcriptome.

Individual expression trajectories for FDR-significant transcripts (FDR < 0.05) in the pooled data set that did not meet the $|log_2FC| > 1$ threshold. Each line represents a single participant, connecting PRE and POST expression values.

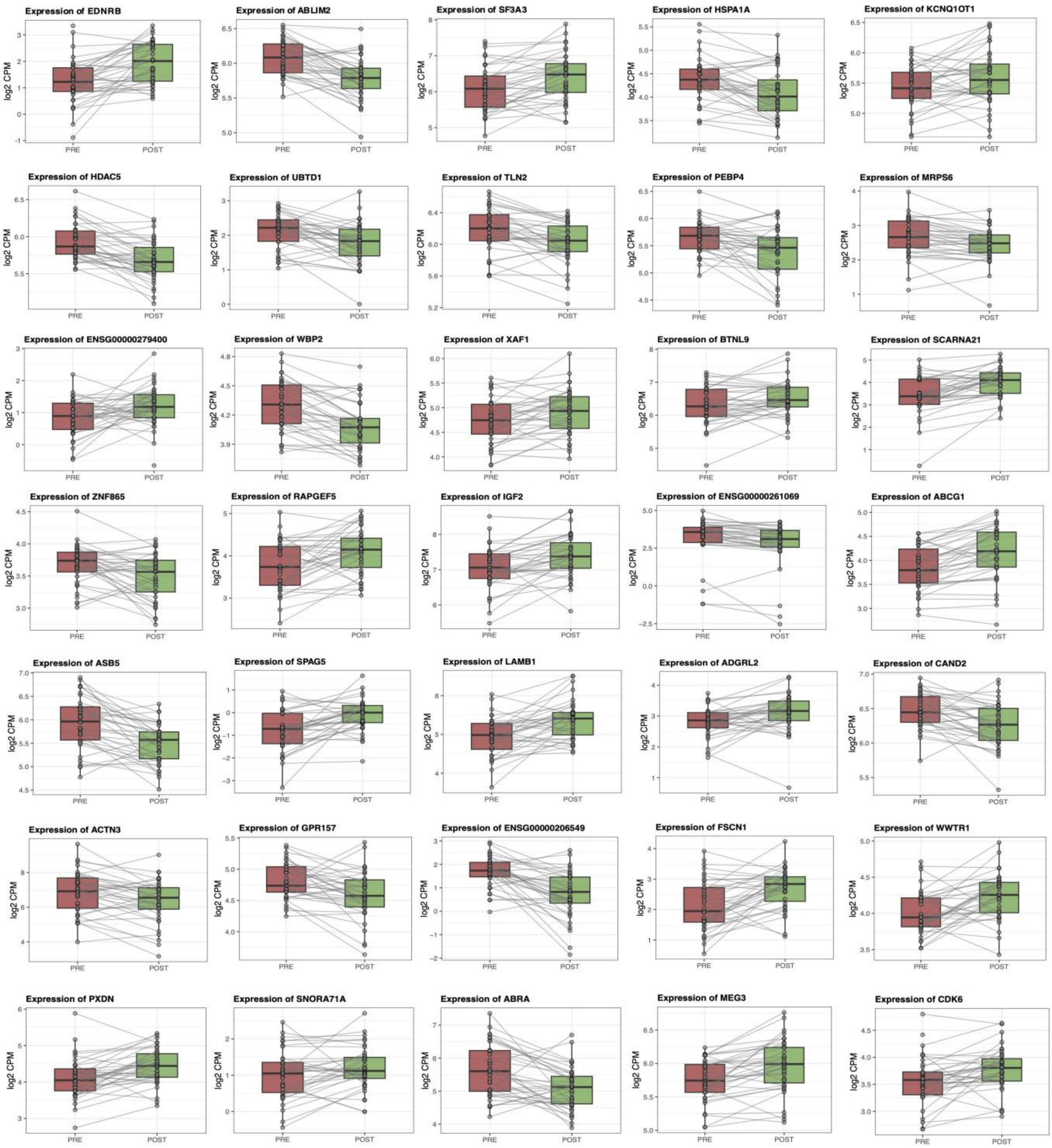

Continuation of Supplementary Figure S4

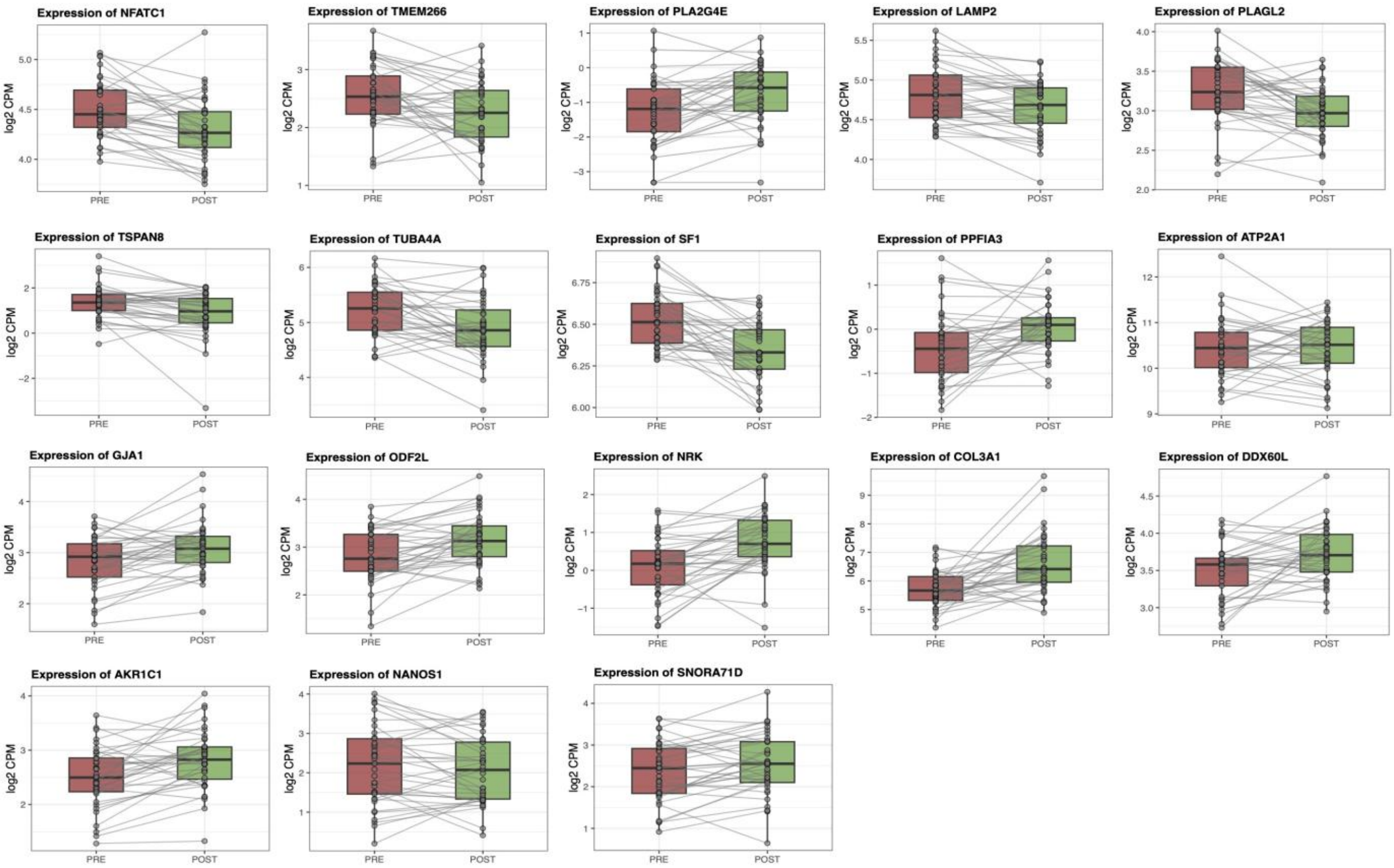

Supplementary Figure S5. Integrated analysis of transcriptomic responses in All-PRE and All-POST samples.

Heatmap of the top 200 genes ranked by FDR, showing z-score normalized expression values per gene across samples. Hierarchical clustering was applied to both genes and samples. Samples segregate primarily by timeframe, indicating a transcriptional shift following intervention. Annotation bars indicate timeframe (PRE vs. POST), sex, age group, intervention group, and muscle fibre composition.

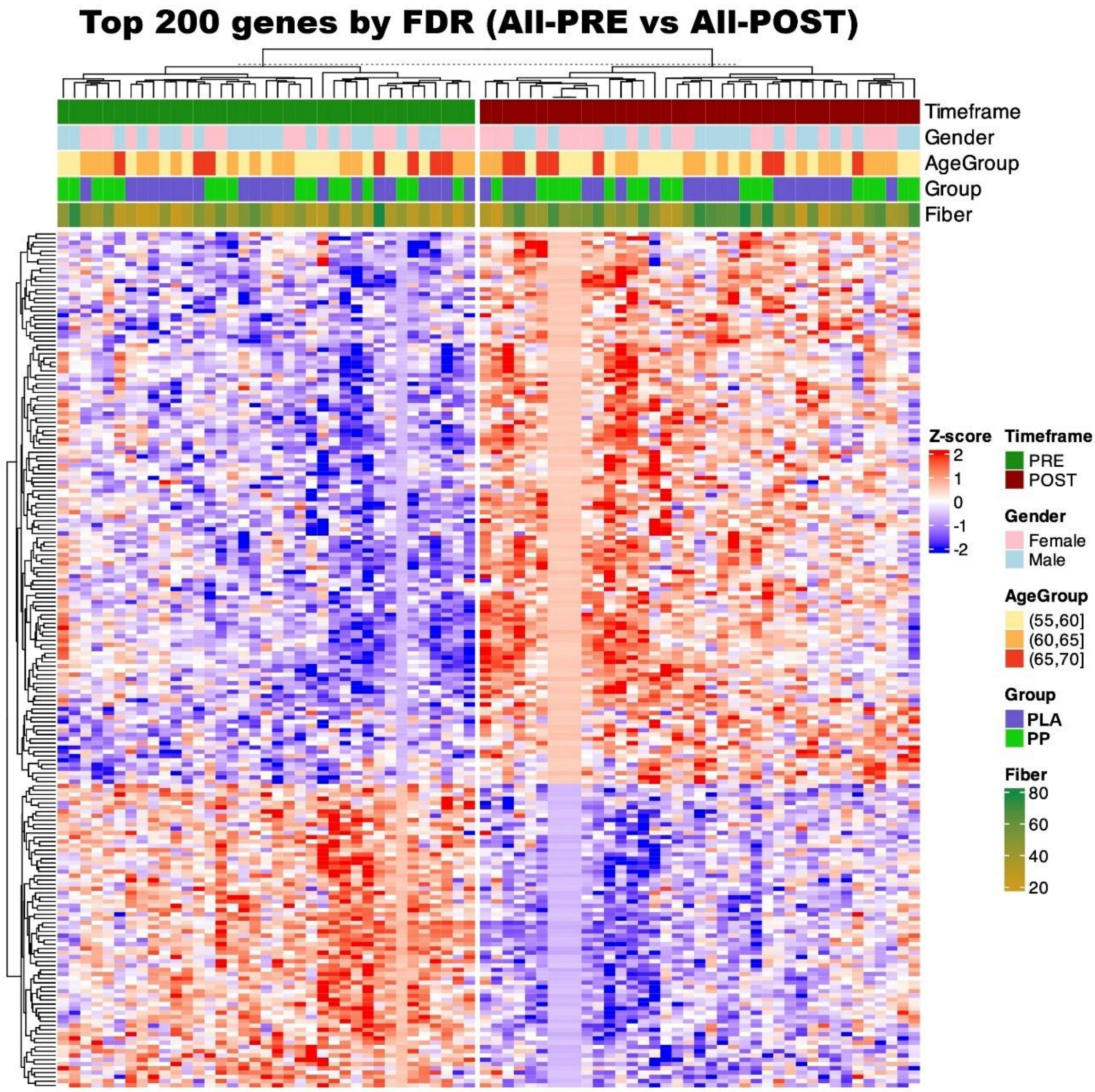

Supplementary Figure S6. Gene set enrichment analysis (GSEA) of the pooled PRE–POST skeletal muscle transcriptomic response.

(A) Enrichment plots for selected Hallmark gene sets identified by GSEA, showing the distribution of gene-set members across the ranked transcriptome. (B) Bubble plot of normalized enrichment scores (NES) for Hallmark gene sets. Dot size reflects gene-set size and colour indicates the FDR-adjusted P value. Positive NES values indicate gene sets enriched among transcripts with higher expression at POST relative to PRE, whereas negative NES values indicate gene sets enriched among transcripts with higher expression at PRE relative to POST.

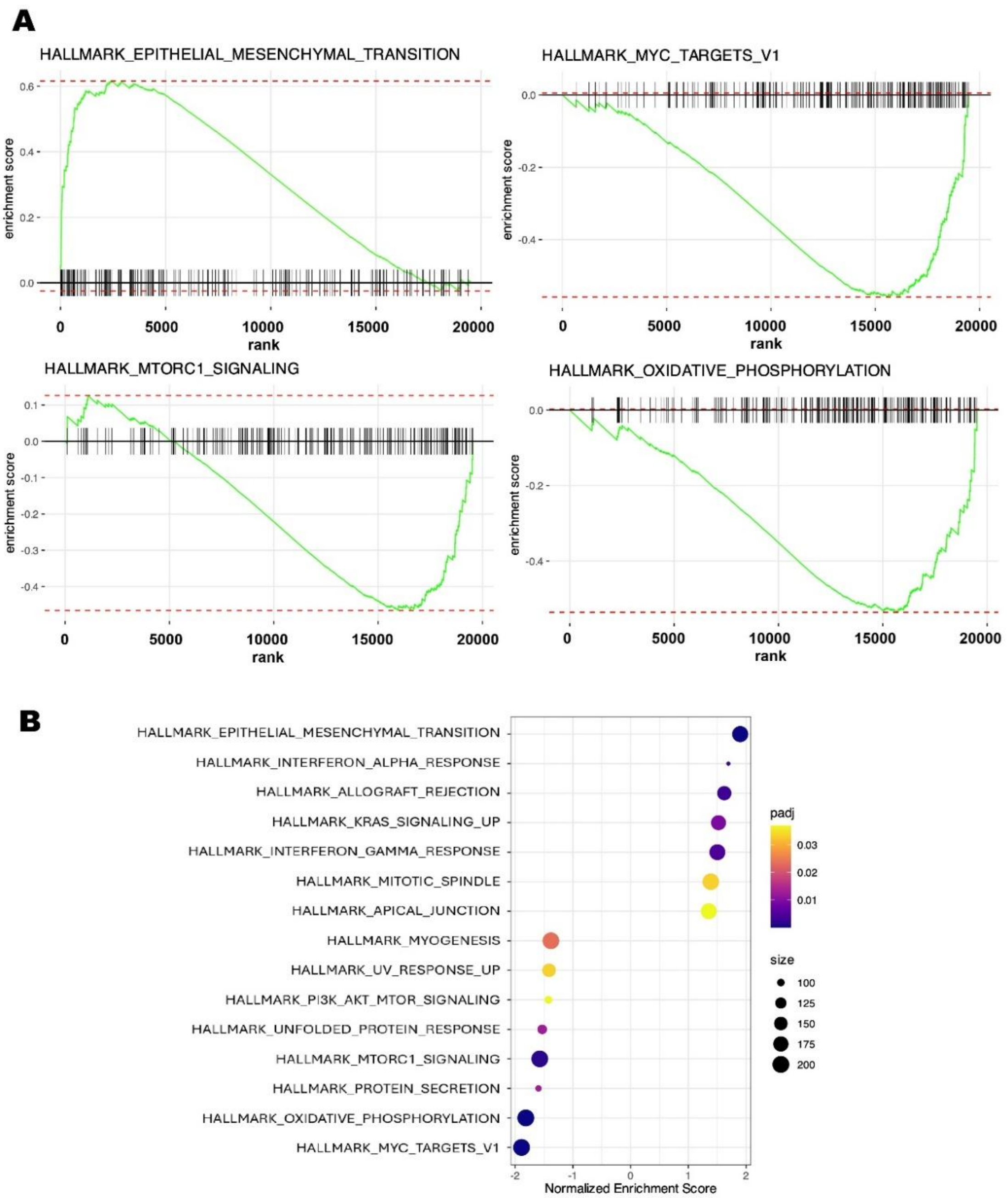

Supplement: Supplementary file 1 [file JCSM-17-e70391-s001.pdf]
