# Supplementary material for: Skeletal Muscle Transcriptomic Responses to Concurrent Training With Polyphenols in Ageing Adults: A Randomized Trial
Source: J Cachexia Sarcopenia Muscle. 2026 Sep 25;17(5):e70391. doi: 10.1002/jcsm.70391 (PMC13613424; doi:10.1002/jcsm.70391)
Supplement: Supplementary file 2 [file JCSM-17-e70391-s002.pdf]

Supplementary Table S1. FDR-significant transcripts in the main differential-expression analyses.

Comparison	Gene symbol	Ensembl ID	Gene name	log2FC	logCPM	LR	P value	FDR	|log2FC| > 1	Combined criterion
Baseline: PP-PRE vs PLA-PRE	RPS3AP46	ENSG00000226132	RPS3A pseudogene 46 [Source:HGNC Symbol;Acc:HGNC:36338]	0.156	-3.104	26.296	2.930E-07	6.797E-03	No	No
Baseline: PP-PRE vs PLA-PRE	HMOX1	ENSG00000100292	heme oxygenase 1 [Source:HGNC Symbol;Acc:HGNC:5013]	1.156	0.460	24.017	9.550E-07	1.108E-02	Yes	Yes
Baseline: PP-PRE vs PLA-PRE		ENSG00000240093	novel transcript	0.733	-2.662	22.651	1.940E-06	1.502E-02	No	No
Group × time interaction	LINC03078	ENSG00000231412	long intergenic non-protein coding RNA 3078 [Source:HGNC Symbol;Acc:HGNC:56665]	0.788	-3.064	58.102	2.490E-14	5.770E-10	No	No
Group × time interaction		ENSG00000276532	Metazoan signal recognition particle RNA [Source:RFAM;Acc:RF00017]	1.018	-3.058	53.442	2.660E-13	3.090E-09	Yes	Yes
Group × time interaction		ENSG00000248452	zinc finger protein 248 (ZNF248) pseudogene	-1.862	-3.067	50.974	9.360E-13	7.240E-09	Yes	Yes
Group × time interaction	GML	ENSG00000104499	glycosylphosphatidylinositol anchored molecule like [Source:HGNC Symbol;Acc:HGNC:4375]	0.139	-2.886	47.708	4.950E-12	2.600E-08	No	No
Group × time interaction		ENSG00000267623	cyclin Y-like pseudogene	-2.521	-2.899	47.464	5.600E-12	2.600E-08	Yes	Yes
Group × time interaction		ENSG00000265394	novel transcript, antisense to EFCAB5	-0.721	-2.954	41.869	9.760E-11	3.770E-07	No	No
Group × time interaction	NA	ENSG00000203386	NA	-2.739	-2.813	38.281	6.130E-10	2.030E-06	Yes	Yes
Group × time interaction	FAM157C	ENSG00000260528	family with sequence similarity 157 member C [Source:HGNC Symbol;Acc:HGNC:34081]	0.040	-1.061	37.803	7.820E-10	2.270E-06	No	No
Group × time interaction		ENSG00000233902	HLA complex group 4 (HCG4) pseudogene	3.019	-2.863	32.109	1.460E-08	3.760E-05	Yes	Yes

Group × time interaction	HTR3B	ENSG00000149305	5-hydroxytryptamine receptor 3B [Source:HGNC Symbol;Acc:HGNC:5298]	-0.212	-2.718	27.685	1.430E-07	3.050E-04	No	No
Group × time interaction	IGHVIII-76-1	ENSG00000253310	immunoglobulin heavy variable (III)-76-1 (pseudogene) [Source:HGNC Symbol;Acc:HGNC:5708]	3.850	-2.884	27.661	1.450E-07	3.050E-04	Yes	Yes
Group × time interaction	IGHG1	ENSG00000211896	immunoglobulin heavy constant gamma 1 (G1m marker) [Source:HGNC Symbol;Acc:HGNC:5525]	-0.906	-0.328	24.440	7.660E-07	1.482E-03	No	No
Group × time interaction	KERA	ENSG00000139330	keratocan [Source:HGNC Symbol;Acc:HGNC:6309]	-2.407	-2.911	22.167	2.500E-06	4.461E-03	Yes	Yes
Group × time interaction		ENSG00000227632	pseudogene similar to part of CDC42 binding protein kinase beta (DMPK-like) CDC42BPB	-2.950	-2.585	17.547	2.800E-05	4.646E-02	Yes	Yes
Pooled paired PRE vs POST	LOXHD1	ENSG00000167210	lipoxygenase homology PLAT domains 1 [Source:HGNC Symbol;Acc:HGNC:26521]	1.043	2.088	39.056	4.120E-10	9.550E-06	Yes	Yes
Pooled paired PRE vs POST	MYH1	ENSG00000109061	myosin heavy chain 1 [Source:HGNC Symbol;Acc:HGNC:7567]	-1.303	11.514	32.747	1.050E-08	1.218E-04	Yes	Yes
Pooled paired PRE vs POST	EDNRB	ENSG00000136160	endothelin receptor type B [Source:HGNC Symbol;Acc:HGNC:3180]	0.848	1.844	26.539	2.580E-07	1.998E-03	No	No
Pooled paired PRE vs POST	LOX	ENSG00000113083	lysyl oxidase [Source:HGNC Symbol;Acc:HGNC:6664]	1.212	0.172	25.762	3.860E-07	2.120E-03	Yes	Yes
Pooled paired PRE vs POST	ABLIM2	ENSG00000163995	actin binding LIM protein family member 2 [Source:HGNC Symbol;Acc:HGNC:19195]	-0.312	5.961	25.438	4.570E-07	2.120E-03	No	No
Pooled paired PRE vs POST	SF3A3	ENSG00000183431	splicing factor 3a subunit 3 [Source:HGNC Symbol;Acc:HGNC:10767]	0.461	6.401	24.625	6.960E-07	2.693E-03	No	No
Pooled paired PRE vs POST	HSPA1A	ENSG00000204389	heat shock protein family A (Hsp70) member 1A [Source:HGNC Symbol;Acc:HGNC:5232]	-0.392	4.323	22.286	2.350E-06	7.786E-03	No	No

Pooled paired PRE vs POST	CLLU1	ENSG00000257127	chronic lymphocytic leukemia up-regulated 1 [Source:HGNC Symbol;Acc:HGNC:29841]	3.102	-2.650	20.554	5.800E-06	1.551E-02	Yes	Yes
Pooled paired PRE vs POST	KCNQ1OT1	ENSG00000269821	KCNQ1 opposite strand/antisense transcript 1 [Source:HGNC Symbol;Acc:HGNC:6295]	0.299	5.571	20.483	6.020E-06	1.551E-02	No	No
Pooled paired PRE vs POST	HDAC5	ENSG00000108840	histone deacetylase 5 [Source:HGNC Symbol;Acc:HGNC:14068]	-0.246	5.827	19.422	1.050E-05	2.111E-02	No	No
Pooled paired PRE vs POST	UBTD1	ENSG00000165886	ubiquitin domain containing 1 [Source:HGNC Symbol;Acc:HGNC:25683]	-0.510	2.059	19.343	1.090E-05	2.111E-02	No	No
Pooled paired PRE vs POST	TLN2	ENSG00000171914	talin 2 [Source:HGNC Symbol;Acc:HGNC:15447]	-0.224	6.125	19.526	9.930E-06	2.111E-02	No	No
Pooled paired PRE vs POST	PEBP4	ENSG00000134020	phosphatidylethanolamine binding protein 4 [Source:HGNC Symbol;Acc:HGNC:28319]	-0.336	5.574	18.758	1.480E-05	2.152E-02	No	No
Pooled paired PRE vs POST	PRND	ENSG00000171864	prion like protein doppel [Source:HGNC Symbol;Acc:HGNC:15748]	2.603	-1.060	18.832	1.430E-05	2.152E-02	Yes	Yes
Pooled paired PRE vs POST	MRPS6	ENSG00000243927	mitochondrial ribosomal protein S6 [Source:HGNC Symbol;Acc:HGNC:14051]	-0.321	2.649	18.909	1.370E-05	2.152E-02	No	No
Pooled paired PRE vs POST	NA	ENSG00000279400	NA	0.640	1.153	19.118	1.230E-05	2.152E-02	No	No
Pooled paired PRE vs POST	WBP2	ENSG00000132471	WW domain binding protein 2 [Source:HGNC Symbol;Acc:HGNC:12738]	-0.224	4.217	18.229	1.960E-05	2.668E-02	No	No
Pooled paired PRE vs POST	XAF1	ENSG00000132530	XIAP associated factor 1 [Source:HGNC Symbol;Acc:HGNC:30932]	0.316	4.888	17.821	2.430E-05	2.668E-02	No	No
Pooled paired PRE vs POST	BTNL9	ENSG00000165810	butyrophilin like 9 [Source:HGNC Symbol;Acc:HGNC:24176]	0.365	6.510	17.992	2.220E-05	2.668E-02	No	No
Pooled paired PRE vs POST		ENSG00000206567	novel transcript	1.075	-0.703	17.979	2.230E-05	2.668E-02	Yes	Yes

Pooled paired PRE vs POST	SCARNA21	ENSG00000252835	small Cajal body-specific RNA 21 [Source:HGNC Symbol;Acc:HGNC:32579]	0.480	3.933	17.751	2.520E-05	2.668E-02	No	No
Pooled paired PRE vs POST	ZNF865	ENSG00000261221	zinc finger protein 865 [Source:HGNC Symbol;Acc:HGNC:38705]	-0.305	3.626	17.742	2.530E-05	2.668E-02	No	No
Pooled paired PRE vs POST	RAPGEF5	ENSG00000136237	Rap guanine nucleotide exchange factor 5 [Source:HGNC Symbol;Acc:HGNC:16862]	0.450	4.051	17.249	3.280E-05	3.082E-02	No	No
Pooled paired PRE vs POST	IGF2	ENSG00000167244	insulin like growth factor 2 [Source:HGNC Symbol;Acc:HGNC:5466]	0.427	7.344	17.265	3.250E-05	3.082E-02	No	No
Pooled paired PRE vs POST		ENSG00000261069	novel transcript, SNORD host	-0.422	3.380	17.225	3.320E-05	3.082E-02	No	No
Pooled paired PRE vs POST	ABCG1	ENSG00000160179	ATP binding cassette subfamily G member 1 [Source:HGNC Symbol;Acc:HGNC:73]	0.290	4.093	17.109	3.530E-05	3.149E-02	No	No
Pooled paired PRE vs POST	ASB5	ENSG00000164122	ankyrin repeat and SOCS box containing 5 [Source:HGNC Symbol;Acc:HGNC:17180]	-0.389	5.802	17.011	3.720E-05	3.193E-02	No	No
Pooled paired PRE vs POST	SPAG5	ENSG00000076382	sperm associated antigen 5 [Source:HGNC Symbol;Acc:HGNC:13452]	0.867	-0.235	16.610	4.590E-05	3.395E-02	No	No
Pooled paired PRE vs POST	LAMB1	ENSG00000091136	laminin subunit beta 1 [Source:HGNC Symbol;Acc:HGNC:6486]	0.421	5.267	16.573	4.680E-05	3.395E-02	No	No
Pooled paired PRE vs POST	ADGRL2	ENSG00000117114	adhesion G protein-coupled receptor L2 [Source:HGNC Symbol;Acc:HGNC:18582]	0.429	3.067	16.807	4.140E-05	3.395E-02	No	No
Pooled paired PRE vs POST	CAND2	ENSG00000144712	cullin associated and neddylation dissociated 2 (putative) [Source:HGNC Symbol;Acc:HGNC:30689]	-0.265	6.395	16.693	4.390E-05	3.395E-02	No	No
Pooled paired PRE vs POST	ACTN3	ENSG00000248746	actinin alpha 3 [Source:HGNC Symbol;Acc:HGNC:165]	-0.683	7.108	16.703	4.370E-05	3.395E-02	No	No

Pooled paired PRE vs POST	GPR157	ENSG00000180758	G protein-coupled receptor 157 [Source:HGNC Symbol;Acc:HGNC:23687]	-0.282	4.735	16.425	5.060E-05	3.453E-02	No	No
Pooled paired PRE vs POST		ENSG00000206549	novel protein identical to PRSS50	-0.752	1.449	16.442	5.020E-05	3.453E-02	No	No
Pooled paired PRE vs POST	FSCN1	ENSG00000075618	fascin actin-bundling protein 1 [Source:HGNC Symbol;Acc:HGNC:11148]	0.612	2.629	16.312	5.370E-05	3.561E-02	No	No
Pooled paired PRE vs POST	WWTR1	ENSG00000018408	WW domain containing transcription regulator 1 [Source:HGNC Symbol;Acc:HGNC:24042]	0.288	4.160	16.235	5.590E-05	3.606E-02	No	No
Pooled paired PRE vs POST	PXDN	ENSG00000130508	peroxidasin [Source:HGNC Symbol;Acc:HGNC:14966]	0.388	4.378	16.047	6.180E-05	3.773E-02	No	No
Pooled paired PRE vs POST	SNORA71A	ENSG00000225091	small nucleolar RNA, H/ACA box 71A [Source:HGNC Symbol;Acc:HGNC:10232]	0.467	1.224	16.068	6.110E-05	3.773E-02	No	No
Pooled paired PRE vs POST	ABRA	ENSG00000174429	actin binding Rho activating protein [Source:HGNC Symbol;Acc:HGNC:30655]	-0.573	5.568	15.957	6.480E-05	3.800E-02	No	No
Pooled paired PRE vs POST	MEG3	ENSG00000214548	maternally expressed 3 [Source:HGNC Symbol;Acc:HGNC:14575]	0.240	5.900	15.936	6.550E-05	3.800E-02	No	No
Pooled paired PRE vs POST	CDK6	ENSG00000105810	cyclin dependent kinase 6 [Source:HGNC Symbol;Acc:HGNC:1777]	0.314	3.734	15.494	8.280E-05	4.512E-02	No	No
Pooled paired PRE vs POST	NFATC1	ENSG00000131196	nuclear factor of activated T cells 1 [Source:HGNC Symbol;Acc:HGNC:7775]	-0.242	4.434	15.469	8.390E-05	4.512E-02	No	No
Pooled paired PRE vs POST	TMEM266	ENSG00000169758	transmembrane protein 266 [Source:HGNC Symbol;Acc:HGNC:26763]	-0.424	2.496	15.389	8.750E-05	4.512E-02	No	No
Pooled paired PRE vs POST	PLA2G4E	ENSG00000188089	phospholipase A2 group IVE [Source:HGNC Symbol;Acc:HGNC:24791]	0.837	-0.743	15.456	8.440E-05	4.512E-02	No	No
Pooled paired PRE vs POST	RAB1AP1	ENSG00000229023	RAB1A pseudogene 1 [Source:HGNC Symbol;Acc:HGNC:54644]	1.401	-1.240	15.411	8.650E-05	4.512E-02	Yes	Yes

Pooled paired PRE vs POST	LAMP2	ENSG00000005893	lysosomal associated membrane protein 2 [Source:HGNC Symbol;Acc:HGNC:6501]	-0.215	4.779	15.149	9.930E-05	4.623E-02	No	No
Pooled paired PRE vs POST	PLAGL2	ENSG00000126003	PLAG1 like zinc finger 2 [Source:HGNC Symbol;Acc:HGNC:9047]	-0.310	3.154	15.178	9.780E-05	4.623E-02	No	No
Pooled paired PRE vs POST	TSPAN8	ENSG00000127324	tetraspanin 8 [Source:HGNC Symbol;Acc:HGNC:11855]	-0.546	1.367	15.159	9.880E-05	4.623E-02	No	No
Pooled paired PRE vs POST	TUBA4A	ENSG00000127824	tubulin alpha 4a [Source:HGNC Symbol;Acc:HGNC:12407]	-0.381	5.153	15.152	9.920E-05	4.623E-02	No	No
Pooled paired PRE vs POST	SF1	ENSG00000168066	splicing factor 1 [Source:HGNC Symbol;Acc:HGNC:12950]	-0.191	6.448	15.102	1.018E-04	4.623E-02	No	No
Pooled paired PRE vs POST	PPFIA3	ENSG00000177380	PTPRF interacting protein alpha 3 [Source:HGNC Symbol;Acc:HGNC:9247]	0.736	-0.025	15.070	1.036E-04	4.623E-02	No	No
Pooled paired PRE vs POST	ATP2A1	ENSG00000196296	ATPase sarcoplasmic/endoplasmic reticulum Ca2+ transporting 1 [Source:HGNC Symbol;Acc:HGNC:811]	-0.309	10.561	15.104	1.017E-04	4.623E-02	No	No
Pooled paired PRE vs POST	GJA1	ENSG00000152661	gap junction protein alpha 1 [Source:HGNC Symbol;Acc:HGNC:4274]	0.366	3.045	14.965	1.095E-04	4.795E-02	No	No
Pooled paired PRE vs POST	ODF2L	ENSG00000122417	outer dense fiber of sperm tails 2 like [Source:HGNC Symbol;Acc:HGNC:29225]	0.371	3.087	14.721	1.246E-04	4.992E-02	No	No
Pooled paired PRE vs POST	NRK	ENSG00000123572	Nik related kinase [Source:HGNC Symbol;Acc:HGNC:25391]	0.624	0.675	14.683	1.272E-04	4.992E-02	No	No
Pooled paired PRE vs POST	COL3A1	ENSG00000168542	collagen type III alpha 1 chain [Source:HGNC Symbol;Acc:HGNC:2201]	0.714	6.590	14.816	1.185E-04	4.992E-02	No	No
Pooled paired PRE vs POST	DDX60L	ENSG00000181381	DExD/H-box 60 like [Source:HGNC Symbol;Acc:HGNC:26429]	0.297	3.662	14.655	1.291E-04	4.992E-02	No	No

Pooled paired PRE vs POST	AKR1C1	ENSG00000187134	aldo-keto reductase family 1 member C1 [Source:HGNC Symbol;Acc:HGNC:384]	0.385	2.769	14.778	1.209E-04	4.992E-02	No	No
Pooled paired PRE vs POST	NANOS1	ENSG00000188613	nanos C2HC-type zinc finger 1 [Source:HGNC Symbol;Acc:HGNC:23044]	-0.418	2.419	14.683	1.272E-04	4.992E-02	No	No
Pooled paired PRE vs POST	SNORA71D	ENSG00000200354	small nucleolar RNA, H/ACA box 71D [Source:HGNC Symbol;Acc:HGNC:32657]	0.379	2.600	14.780	1.208E-04	4.992E-02	No	No

Note: All transcripts reaching a Benjamini–Hochberg false discovery rate (FDR) ≤ 0.05 are reported. The $|\log_2 FC| > 1$ column identifies transcripts additionally exceeding the prespecified effect-size threshold. The combined criterion denotes FDR ≤ 0.05 together with $|\log_2 FC| > 1$. In the baseline comparison (PP-PRE vs PLA-PRE), 3 transcripts reached FDR ≤ 0.05, of which 1 also met the combined criterion. In the group × time interaction analysis, 14 transcripts reached FDR ≤ 0.05, of which 8 met the combined criterion. In the pooled paired PRE–POST analysis, 60 transcripts reached FDR ≤ 0.05, of which 7 met the combined criterion. $\log_2 FC$, estimated $\log_2$ fold change from the corresponding differential-expression model; logCPM, average $\log_2$ counts per million; LR, likelihood-ratio statistic; FDR, Benjamini–Hochberg false discovery rate; PP, polyphenol group; PLA, placebo group; PRE, pre-intervention; POST, post-intervention.

Supplementary Table S2. Sample metadata, RNA quality-control metrics, sequencing characteristics, and mapping statistics.

Sample-id		*Metadata*							*RNA quality control*			*Raw sequence data*		*Mapping results*				Comments
		**Individual**	**Timeframe**	**Group**	**Sex**	**Age**	**Slow fibers (%)**	**Fast fibers (%)**	**Concentration (ng/uL)**	**RIN**	**DV200 (%)**	**No of fragments (in million)**	**No of bases (in Gb)**	**No of mapped fragments (in million)**	**Proportion of sequenced fragments (%)**	**No of fragments used for expression quantification (in million)**	**Proportion of fragments used for expression quantification (%)**	
1-	S12946Nr1	101	PRE	Polyphenol	Male	69	45.511	54.489	24.6	1.0	31.0	38.447	7.506	34.694	90.2	13.404	38.6	Outlier (z-score=-4.61)
2	S12946Nr10	107	POST	Polyphenol	Female	66	56.823	43.177	35.3	1.0	23.9	93.530	17.466	87.922	94.0	30.570	34.8	-
3	S12946Nr11	109	PRE	Placebo	Male	61	72.699	27.301	107.6	1.3	29.5	50.773	9.577	47.540	93.6	11.796	24.8	-
4	S12946Nr12	109	POST	Placebo	Male	61	27.376	72.624	19.6	1.3	36.0	60.143	11.582	57.504	95.6	25.744	44.8	-
5	S12946Nr13	111	PRE	Placebo	Female	56	65.825	34.175	76.5	1.0	25.9	85.262	16.255	79.698	93.5	27.083	34.0	-
6	S12946Nr14	111	POST	Placebo	Female	56	47.072	52.928	122.2	1.0	29.5	94.098	18.131	87.889	93.4	31.591	35.9	-
7	S12946Nr15	114	PRE	Polyphenol	Female	55	43.353	56.647	48.1	1.1	36.0	111.067	21.587	105.461	95.0	49.104	46.6	-
8	S12946Nr16	114	POST	Polyphenol	Female	55	50.984	49.016	65.1	1.2	26.5	112.514	21.746	106.559	94.7	43.826	41.1	-
9	S12946Nr17	117	PRE	Polyphenol	Male	58	57.021	42.979	163.8	1.0	22.8	44.507	8.483	41.969	94.3	12.886	30.7	-

10	S12946Nr19	119	PRE	Placebo	Male	59	59.102	40.898	98.4	1.2	33.0	74.126	14.315	70.794	95.5	31.533	44.5	-
11	S12946Nr2	101	POST	Poly-phenol	Male	69	31.877	68.123	75.8	1.1	33.8	57.626	11.198	54.635	94.8	22.686	41.5	-
12	S12946Nr20	119	POST	Placebo	Male	59	58.482	41.518	53.1	1.2	34.0	75.805	14.368	71.920	94.9	30.108	41.9	-
13	S12946Nr21	121	PRE	Placebo	Female	63	60.462	39.538	119.8	1.0	21.5	65.882	12.775	62.293	94.6	17.919	28.8	-
14	S12946Nr22	121	POST	Placebo	Female	63	67.476	32.524	151.3	1.0	15.9	51.293	9.829	48.120	93.8	13.883	28.9	-
15	S12946Nr23	123	PRE	Placebo	Female	58	68.597	31.403	171.4	1.1	14.6	42.956	8.026	39.837	92.7	10.542	26.5	-
16	S12946Nr24	123	POST	Placebo	Female	58	49.284	50.716	146.2	1.1	13.2	40.320	7.574	37.663	93.4	10.442	27.7	-
17	S12946Nr25	125	PRE	Poly-phenol	Male	60	68.575	31.425	114.7	1.0	10.6	55.917	10.612	52.197	93.3	14.147	27.1	-
18	S12946Nr26	125	POST	Poly-phenol	Male	60	70.921	29.079	82.8	1.1	14.4	62.176	11.838	58.157	93.5	16.829	28.9	-
19	S12946Nr27	126	PRE	Placebo	Male	65	49.955	50.045	48.8	1.2	19.5	85.034	16.072	79.223	93.2	25.809	32.6	-
20	S12946Nr28	126	POST	Placebo	Male	65	49.008	50.992	207.4	1.2	9.2	76.861	14.230	70.483	91.7	16.253	23.1	-
21	S12946Nr29	127	PRE	Poly-phenol	Male	56	68.661	31.339	176.6	1.0	14.1	87.313	16.163	79.857	91.5	21.657	27.1	-
22	S12946Nr3	102	PRE	Poly-phenol	Female	62	62.529	37.471	117.0	1.4	22.1	78.809	14.677	73.084	92.7	24.494	33.5	-
23	S12946Nr30	127	POST	Poly-phenol	Male	56	59.644	40.356	154.6	1.0	11.3	86.240	15.860	78.222	90.7	19.639	25.1	-
24	S12946Nr31	128	PRE	Placebo	Male	60	49.571	50.429	139.5	1.0	11.6	74.427	13.453	67.838	91.1	16.045	23.7	-
25	S12946Nr32	128	POST	Placebo	Male	60	38.324	61.676	233.5	1.1	15.0	92.469	17.455	85.874	92.9	25.109	29.2	-
26	S12946Nr33	129	PRE	Poly-phenol	Male	66	71.143	28.857	86.7	1.0	10.2	10.200	8.963	43.529	92.5	12.831	29.5	-

27	S12946Nr34	129	POST	Poly-phenol	Male	66	64.474	35.526	71.1	1.0	12.9	52.641	10.051	48.958	93.0	16.945	34.6	-
28	S12946Nr35	131	PRE	Placebo	Male	61	76.196	23.804	191.8	1.1	10.4	53.261	9.848	48.912	91.8	11.080	22.7	-
29	S12946Nr36	131	POST	Placebo	Male	61	75.828	24.172	146.5	1.1	10.0	61.651	11.222	56.191	91.1	13.819	24.6	-
30	S12946Nr37	132	PRE	Poly-phenol	Male	58	46.152	53.848	130.9	1.0	19.9	51.120	9.846	47.544	93.0	16.931	35.6	-
31	S12946Nr38	132	POST	Poly-phenol	Male	58	32.806	67.194	139.5	1.0	15.5	47.862	9.112	44.295	92.5	15.886	35.9	-
32	S12946Nr39	134	PRE	Poly-phenol	Female	59	51.947	48.053	99.8	1.1	14.3	54.396	10.366	50.498	92.8	17.149	34.0	-
33	S12946Nr4	102	POST	Poly-phenol	Female	62	34.879	65.121	36.5	1.5	34.3	86.355	16.399	81.402	94.3	35.679	43.8	-
34	S12946Nr40	134	POST	Poly-phenol	Female	59	67.807	32.193	95.3	1.0	14.7	59.594	11.401	55.697	93.5	21.145	38.0	-
35	S12946Nr41	137	PRE	Placebo	Male	55	54.336	45.664	48.4	1.0	22.1	55.065	10.330	50.895	92.4	15.000	29.5	-
36	S12946Nr42	137	POST	Placebo	Male	55	42.029	57.971	61.9	1.0	23.6	64.987	12.212	59.959	92.3	13.542	22.6	-
37	S12946Nr43	138	PRE	Poly-phenol	Male	61	62.955	37.045	98.5	1.1	18.7	57.916	10.942	53.202	91.9	15.470	29.1	-
38	S12946Nr44	138	POST	Poly-phenol	Male	61	21.433	78.567	27.0	1.1	23.6	66.671	12.625	62.009	93.0	21.193	34.2	-
39	S12946Nr45	140	PRE	Placebo	Male	70	75.659	24.341	80.9	1.2	32.4	46.316	8.968	43.816	94.6	14.414	32.9	-
40	S12946Nr46	140	POST	Placebo	Male	70	33.393	66.607	83.3	1.1	27.4	63.111	12.078	59.130	93.7	15.450	26.1	-
41	S12946Nr47	141	PRE	Placebo	Female	58	67.747	32.253	67.3	1.1	21.5	49.746	9.470	46.525	93.5	12.075	26.0	-
42	S12946Nr48	141	POST	Placebo	Female	58	52.475	47.525	53.5	1.1	28.1	56.816	10.927	53.335	93.9	16.007	30.0	-
43	S12946Nr49	144	PRE	Placebo	Female	66	58.588	41.412	53.4	1.0	18.7	71.086	13.614	66.621	93.7	21.368	32.1	-

44	S12946Nr5	104	PRE	Placebo	Female	64	63.356	36.644	150.6	1.0	22.4	34.993	6.653	32.754	93.6	10.158	31.0	-
45	S12946Nr50	144	POST	Placebo	Female	66	49.718	50.282	32.8	1.0	24.2	59.296	11.340	55.620	93.8	18.777	33.8	-
46	S12946Nr51	145	PRE	Poly-phenol	Female	61	76.948	23.052	97.5	1.1	20.1	66.973	12.787	62.154	92.8	16.967	27.3	-
47	S12946Nr52	145	POST	Poly-phenol	Female	61	76.637	23.363	44.0	1.4	33.9	97.596	18.770	90.657	92.9	31.333	34.6	-
48	S12946Nr53	148	PRE	Poly-phenol	Male	57	52.693	47.307	62.3	1.0	21.5	61.358	11.658	57.648	94.0	16.153	28.0	-
49	S12946Nr54	148	POST	Poly-phenol	Male	57	30.724	69.276	28.6	1.0	29.8	52.049	10.010	49.162	94.5	15.842	32.2	-
50	S12946Nr55	149	PRE	Placebo	Male	55	56.107	43.893	63.8	1.1	22.4	50.822	9.687	47.624	93.7	13.563	28.5	-
51	S12946Nr56	149	POST	Placebo	Male	55	34.039	65.961	59.3	1.1	24.5	65.713	12.546	61.611	93.8	14.573	23.7	-
52	S12946Nr57	153	PRE	Poly-phenol	Male	60	25.445	74.555	62.4	1.0	14.1	41.607	7.837	38.281	92.0	9.443	4.7	-
53	S12946Nr58	153	POST	Poly-phenol	Male	60	57.578	42.422	64.8	1.0	24.3	78.221	14.986	73.057	93.4	20.396	27.9	-
54	S12946Nr59	155	POST	Poly-phenol	Female	55	49.217	50.783	67.3	1.1	23.7	52.753	10.164	49.829	94.5	15.624	31.4	-
55	S12946Nr6	104	POST	Placebo	Female	64	50.38	49.62	111.1	1.0	21.4	66.262	12.659	62.457	94.3	19.734	31.6	-
56	S12946Nr60	156	PRE	Placebo	Female	61	74.653	25.347	47.0	1.1	27.5	40.663	7.728	38.165	93.9	13.551	35.5	-
57	S12946Nr61	156	POST	Placebo	Female	61	78.18	21.82	62.3	1.0	26.4	88.057	16.974	82.195	93.3	31.535	38.4	-
58	S12946Nr62	157	PRE	Placebo	Male	64	38.948	61.052	99.0	1.0	23.4	63.962	12.328	59.305	92.7	17.840	30.1	-
59	S12946Nr63	157	POST	Placebo	Male	64	37.353	62.647	20.7	1.1	40.8	82.956	16.146	79.168	95.4	26.154	33.0	-
60	S12946Nr64	158	PRE	Placebo	Female	68	21.527	78.473	29.7	1.1	40.4	68.882	13.484	65.791	95.5	24.404	37.1	-

61	S12946Nr65	158	POST	Placebo	Female	68	33.329	66.671	78.7	1.0	18.9	52.588	10.150	49.521	94.2	15.963	32.2	-
62	S12946Nr66	160	PRE	Placebo	Male	67	50.702	49.298	48.5	1.1	31.0	50.954	9.915	48.552	95.3	18.185	37.5	-
63	S12946Nr67	160	POST	Placebo	Male	67	58.181	41.819	99.8	1.0	29.1	41.740	8.073	39.520	94.7	12.807	32.4	-
64	S12946Nr68	161	PRE	Poly-phenol	Female	69	60.905	39.095	166.3	1.1	40.2	54.279	10.607	51.645	95.1	19.010	36.8	-
65	S12946Nr69	161	POST	Poly-phenol	Female	69	24.289	75.711	19.3	1.2	54.2	65.254	12.822	62.420	95.7	28.063	45.0	-
66	S12946Nr7	106	PRE	Placebo	Male	63	77.001	22.999	125.2	1.1	27.9	95.397	17.800	86.972	91.2	30.630	35.2	-
67	S12946Nr70	162	PRE	Placebo	Female	64	56.691	43.309	54.4	1.1	48.1	65.376	12.813	62.193	95.1	26.541	42.7	-
68	S12946Nr71	162	POST	Placebo	Female	64	62.913	37.087	132.3	1.1	30.7	55.950	10.820	53.058	94.8	19.483	36.7	-
69	S12946Nr72	163	PRE	Placebo	Female	60	71.801	28.199	94.7	1.0	36.9	59.430	11.506	56.380	94.9	14.203	25.2	-
70	S12946Nr73	163	POST	Placebo	Female	60	53.474	46.526	136.8	1.0	30.6	54.936	10.706	51.950	94.6	15.565	30.0	-
71	S12946Nr74	165	PRE	Poly-phenol	Female	65	72.817	27.183	72.6	1.0	26.3	55.417	10.810	51.793	93.5	17.276	33.4	-
72	S12946Nr75	165	POST	Poly-phenol	Female	65	59.017	40.983	100.0	1.0	16.9	54.880	10.469	51.326	93.5	13.614	26.5	-
73	S12946Nr76	167	PRE	Poly-phenol	Female	63	42.269	57.731	51.5	1.1	35.8	54.699	10.732	52.268	95.6	21.214	40.6	-
74	S12946Nr77	167	POST	Poly-phenol	Female	63	42.807	57.193	116.2	1.0	38.4	59.754	11.471	56.319	94.3	22.611	40.1	-
75	S12946Nr79	169	POST	Poly-phenol	Female	56	52.894	47.106	143.5	1.1	42.5	55.606	10.901	53.212	95.7	20.277	38.1	-
76	S12946Nr8	106	POST	Placebo	Male	63	62.058	37.942	35.7	1.0	27.2	89.719	17.004	84.067	93.7	30.741	36.6	-
77	S12946Nr9	107	PRE	Poly-phenol	Female	66	71.573	28.427	36.9	1.0	22.4	38.405	7.371	36.543	95.2	12.998	35.6	-

Note: Sample-level metadata and RNA-sequencing quality-control information for skeletal muscle samples collected before (PRE) and after (POST) the training intervention in the polyphenol and placebo groups. The table includes participant characteristics and muscle fibre-type composition, RNA concentration, RNA integrity number (RIN), DV200, raw sequencing depth (number of fragments and bases), mapping statistics, and the number and proportion of mapped fragments used for gene-expression quantification. Comments regarding sample-level quality-control findings and exclusions are also provided.
